# Clinical research capability enhanced for medical undergraduates: an innovative simulation-based clinical research curriculum development

**DOI:** 10.1186/s12909-022-03574-6

**Published:** 2022-07-14

**Authors:** Siyu Yan, Qiao Huang, Jiao Huang, Yu Wang, Xuhui Li, Yongbo Wang, Lisha Luo, Yunyun Wang, Yi Guo, Xiantao Zeng, Yinghui Jin

**Affiliations:** 1 Center for Evidence-Based and Translational Medicine, Zhongnan Hospital of Wuhan University, 169 Donghu Road, Wuchang District, Wuhan, 430071 China; 2 Department of Evidence-Based Medicine and Clinical Epidemiology, Second School of Clinical Medicine, Wuhan University, 169 Donghu Road, Wuchang District, Wuhan, 430071 China

**Keywords:** Curriculum Development, Simulation, Clinical Research, Undergraduate Education, Survey, Physician-scientist

## Abstract

**Background:**

Clinical research has frequently not been taught in a practical way, often resulting in a very didactic approach rendering it not very accessible for medical undergraduates. Simulation can provide an immersive, interactive, and reflective experience and may be applied to the clinical research curriculum.

**Methods:**

A 7-step model, modified from Kern’s six-step approach and Khamis’s stepwise model, was used to develop the curriculum. A questionnaire survey on undergraduates’ attitude towards, knowledge and practice of clinical research and simulation education was conducted to generate a targeted needs assessment. The simulation framework was integrated into the development of educational strategies. Experts were consulted to assess the curriculum prior to implementation.

**Results:**

Talent construction in China needs an innovative capability-enhanced clinical research curriculum. Sixty-six clinical undergraduates in our school completed the survey. 89.39% (59/66) of them hadn’t participated in clinical research, while 93.94% (62/66) would like to conduct clinical trials if possible. 75.76% of respondents didn’t have knowledge of or practical abilities in clinical trials. The mean score for practical ability (2.02 ± 0.92) was lower than that of knowledge (2.20 ± 0.93) (*P* < 0.01). The dimension of case report form got the lowest score among the five dimensions. Participating in clinical research (*P* = 0.04) and learning for themselves (*P* < 0.01) by a few students may have increased the total score. The curriculum was designed to simulate the whole process from protocol writing, registration, ethical approval, implementation, and data analysis to reporting based on one case study, and was divided into two parts to simulate different types of research: randomized controlled trials and observational studies. It was conducted in semesters 5 and 7 respectively, both including 16 sessions. After expert consultation, one session having a 29.01% coefficient of variation was adjusted and replaced. The final simulation class design scenario scripts are provided for reference.

**Conclusions:**

The targeted needs assessment exposed medical undergraduates’ poor knowledge of and abilities in clinical research. This is the first report of a simulation-based clinical research curriculum developed in China, and adds curriculum development and design details to the limited related published studies.

**Supplementary Information:**

The online version contains supplementary material available at 10.1186/s12909-022-03574-6.

## Introduction

The outbreak of the Coronavirus Disease 2019 (COVID-19) epidemic has highlighted the importance of clinical research and revealed the problems caused by non-standard clinical research resulting in a waste of limited resources [1]. The quantity and quality of talents may be an important factor affecting the quality of clinical research. As early as 1979, James Wyngaarden described the physician-scientist as an “endangered species” [2]. Since the 1990s, the National Institute of Health (NIH) in the USA has furnished tremendous concerns for the development of clinical research training [3]. Despite these concerns and efforts, the attrition rate for physician-scientists has not been stopped or even significantly slowed [4]. Currently, there are no physician-scientists (physicians who have substantial protected time for research) in China, and many physicians do not have the skills to understand and carry out clinical research to an international standard [5]. An important barrier has been the absence of systematic training in clinical research in medical education institutions [5].

In our teaching process, we found that although students have achieved good test scores in related theory courses, they have shown poor ability in carrying out real clinical research, confirming Peacock’s findings [6]. Some possible reasons are: firstly, as the growth, complexity and scale of clinical research has increased so has the demand for a higher level of technical expertise to execute it. However, related disciplines are isolated rather than integrated preventing the provision of comprehensive training; secondly, research design courses for undergraduates are often regarded as esoteric [7, 8]. This suggests that a reform of clinical research courses is necessary.

Simulation, as an emerging medical education strategy, injects vitality into the medical education field [9–11]. It can provide an efficient and safe learning environment for clinical teaching and is widely used in teaching both clinical technical and procedural skills and high risk and invasive interventions [12]. However, reports on simulation in research design course are very limited [6, 13–15].

As Gaba said “Simulation is a technique—not a technology—to replace or amplify real experiences with guided experiences that evoke or replicate substantial aspects of the real world in a fully interactive manner” [10]. We believe that simulation of clinical research, such as randomized controlled trials, cohort studies and cross-sectional studies, can also be applicable and beneficial.

In this study, we have explored introducing simulation into the teaching of clinical research, aiming at bridging the gap between knowledge and application and helping students to enhance their capability and confidence to conduct research in the future.

## Methods

**Fig. 1:**
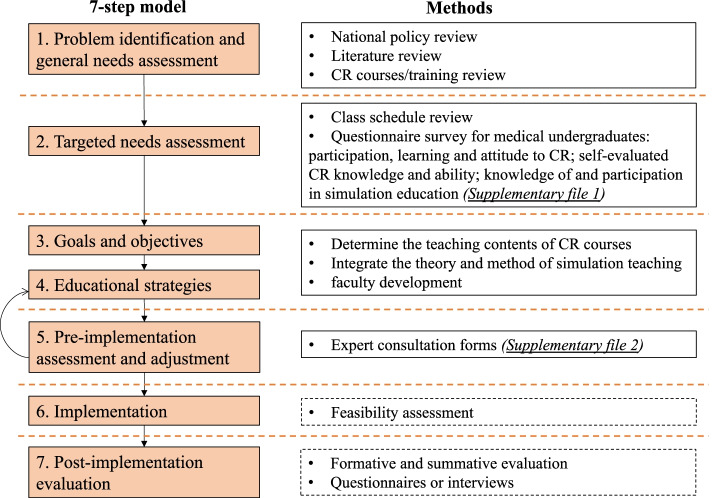
Framework of the 7-step model for simulation-based medical curriculum development and corresponding methods. CR: clinical research

The simulated clinical research curriculum was developed based on our 7-step model which was a modification from the six-step approach in curriculum development for medical education proposed by Kern [16] and the stepwise model proposed by Khamis et al. for simulation-based curriculums development for clinical skills [17]. Figure 1 shows the framework of the 7-step model.

The seven steps include:

- *Step 1: Problem identification and general needs assessment*
  Needs assessment at the international, national, or regional level should be done as the first step, thus we reviewed the national overall planning of medical education, assessed the demand for clinical research talents, and identified the development of clinical research curricula at home and abroad.

- *Step 2: Targeted needs assessment*
  This step is usually done at the institutional level and involves the collection of data on the institution’s targeted learners and learning environment. Our curriculum was designed for the eight-year program medical students, who will obtain a doctor of medicine degree upon graduation [18]. We reviewed their class schedule, and conducted an anonymous online survey (Supplementary file 1) of clinical medicine undergraduates in our school. The questionnaire link was sent to the class groups and students filled it out voluntarily. The questionnaire encompassing self-evaluation of clinical research knowledge and practical ability, included five dimensions and 22 questions, and used a 5-point Likert scale (1 = Know absolutely nothing; 2 = Don’t know; 3 = Know something; 4 = Know a reasonable amount; 5 = Know everything). A pilot study was conducted before the formal survey, in which 20 students from the same medical school were invited to fill the questionnaire. This was then re-administered two weeks later and the test–retest reliability coefficient (intraclass correlation coefficient, ICC) of included items ranged from 0.62 to 0.89, the mean ICC was 0.76 which indicated good reliability. Following this the main questionnaire was administered. Mean, standard deviation (SD) and percentage of score lower than 3 for each question and dimension (the dimension score was the average score of questions in dimension) were calculated for description, and the singed rank test was used to test the difference between knowledge and practical ability. Wilcoxon signed rank test and Kruskal Wallis test were used to test the difference between total scores among respondents’ characteristics. Knowledge of and participation in simulation-based education were also investigated.

- *Step 3: Goals and objectives*
  The goals were set according to the results of general and targeted needs assessment, and the objectives were set to fit in with the course organization, including measurable evaluation of knowledge and skills at individual and team levels.

- *Step 4: Educational strategies*
  The educational strategies included the educational contents, educational methods and faculty development.
  We determined the teaching contents of the clinical research courses by referring to the good clinical practice (GCP) guideline released by the National Medical Products Administration (NMPA) [19], the existing training system for clinical research [20–22] and knowledge and experience from a sufficient number and variety of consultants. Meanwhile, we integrated the theory and simulation teaching methods into the educational contents [23–26].
  To facilitate faculty development, training classes in medical simulation teaching were provided, and a teaching support group was set to facilitate communication among teachers.

- *Step 5: Pre-implementation assessment and adjustment*
  Because the development of simulation curricula is usually resource-intensive, there is a need to ensure the validity of the teaching contents through literature review and consensus input from multiple stakeholders and experts [17]. A five-point Likert scale (1 = Strongly disagree; 2 = Disagree; 3 = Neither agree nor disagree; 4 = Agree; 5 = Strongly agree) based on our developed syllabus was used for expert consultation (Supplementary file 2). It also included space to express disagreements and ideas for supplementation. Mean, SD and coefficients of variation (CV) were calculated for each item. Adjustments were made after discussion of expert opinions.

- *Step 6: Implementation*
  The implementation of the curriculum needs to consider: assessment of institutional capacity and faculty’s expertise, political and administrative support, resources, curriculum administration, identification and addressing of barriers to the curriculum implementation.

- *Step 7: Post-implementation evaluation*
  Formative and summative evaluation will be used for individual assessment and feedback. Questionnaires or interviews for participants will be conducted after curriculum implementation to evaluate satisfaction with and effectiveness of the curriculum.

## Results

### Step 1: Problem identification and general needs assessment

The review of national overall planning of medical education and demands for clinical research talents have shown the need for innovative clinical research courses. Standards for Basic Medical Education in China require medical schools to teach medical research methods throughout the curriculum [27]. The Guiding Opinions on Comprehensively Promoting Health and Health Technology Innovation in 2016 emphasized the need to improve the clinical medicine research system and increase capacity building, and proposed improving clinical medical research across-the-board [28].

The “Guiding Opinions on Accelerating the Innovation and Development of Medical Education” issued by the General Office of the State Council in 2020 stated that “by 2030, a higher-level medical talent training system with Chinese characteristics will be established, and medical scientific research and innovation capabilities will be significantly improved to serve health care” [29].

About 60 universities and research institutes in the United States are establishing or have established degree-based clinical research training programs (such as the Duke University Clinical Research Master’s Program). The Clinical Research Center of NIH provides an extensive clinical research training [20]. The European Union has also formulated a unified training strategy for clinical researchers [21], and created a clinical investigator course learning website [22].

The NMPA has also updated the GCP guideline for clinical drug trials on April 26, 2020 [19]. The Department of Clinical Epidemiology and Clinical Trials was established in Capital Medical University on May 21, 2020 [30]. Before this, no university in China had set up majors in clinical research or clinical trials, and practitioners have obtained relevant knowledge mainly from the training available in the organizations where they work [31]. If we don’t develop systematic training for clinical research in medical education, the shortage of talents may continue to become a conspicuous problem and restrict the development of clinical research in China.

### Step 2: Targeted needs assessment

The review of research training for the eight-year program medical students in our school showed that students had learned medical ethics, medical statistics, epidemiology, and SPSS statistical analysis before the fifth semester (Supplementary file 3: Figure S1). But these totally theoretical courses were presented as separate stand-alone courses which renders integrated practical courses very necessary.

**Fig. 2:**
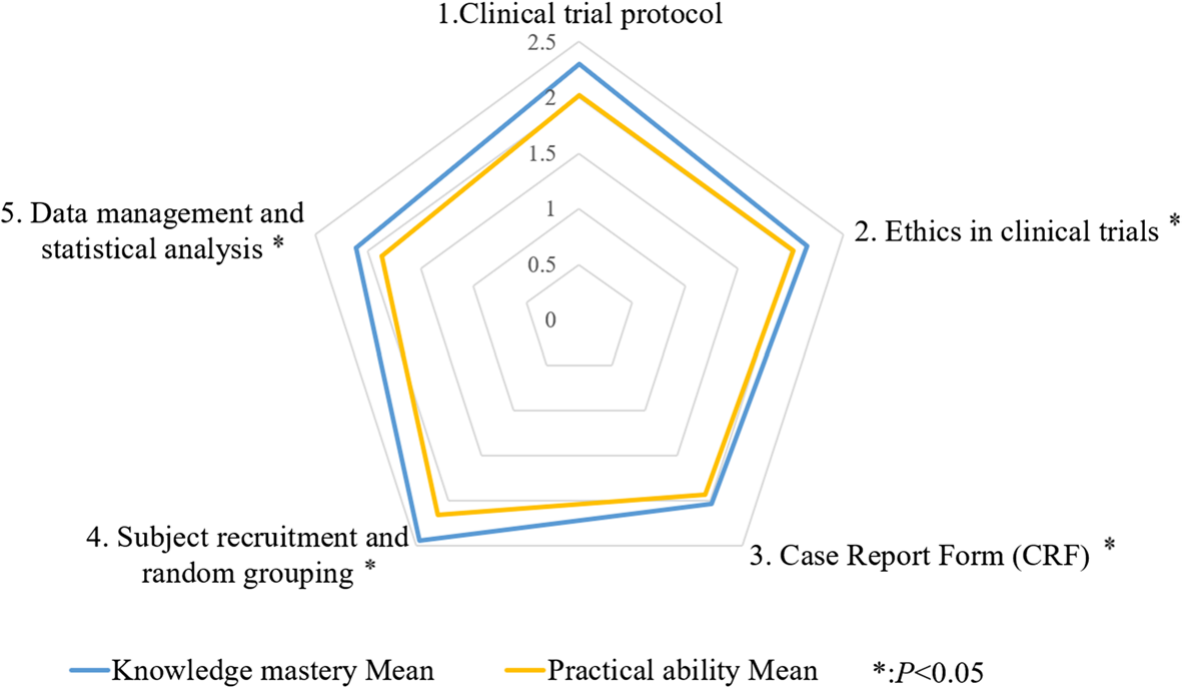
The mean scores of knowledge mastery and practical ability in clinical trials for five dimensions in the survey
  More than 50% of the students had scores lower than 3 in the area of practical abilities in all 22 questions in these 5 dimensions (Table S2). In areas of both knowledge mastery and practical abilities, the question “establishment and management of the clinical trial database” scored the lowest among 22 questions (1.86 ± 0.87 and 1.74 ± 0.97, respectively) (Fig. 3, Table S2).

**Fig. 3:**
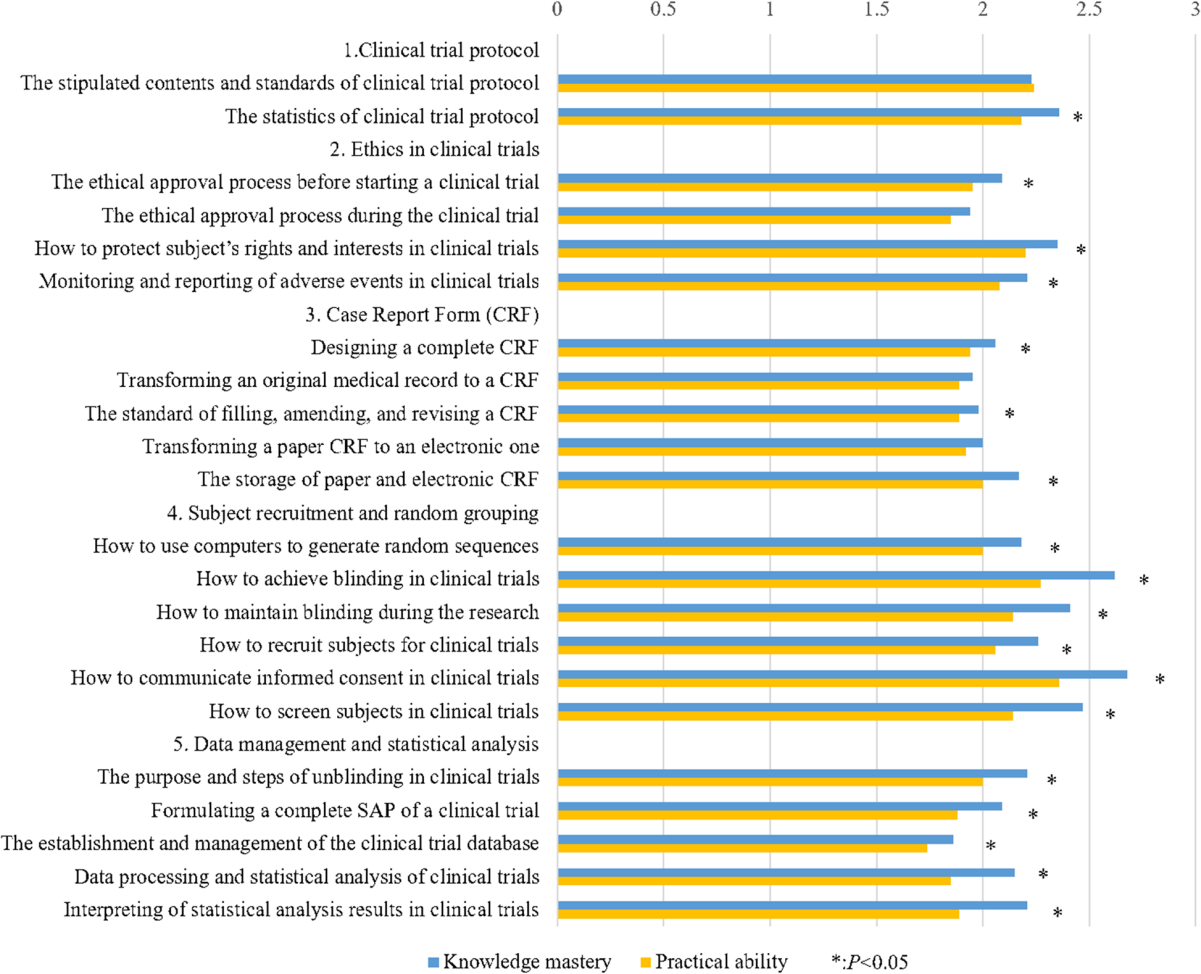
The mean scores of knowledge mastery and practical ability in clinical trials for each question

Results of the questionnaire survey on medical students’ cognition of clinical trials and simulation teaching were as follows:

- *Basic information and attitude*
  A total of 66 clinical medicine students filled out our online questionnaire, and the results are presented in Table S1 (Supplementary file 3). 89.39% (59/66) of them hadn’t participated in clinical research before. 84.85% (56/66) of them hadn’t taken any relevant systematic training in clinical trials. 46.97% (31/66) of them had learned relevant knowledge using their own initiative, and 93.94% (62/66) of them would like to carry out clinical trials if possible.

- *Knowledge of and practical abilities in clinical trials*
  The results of self-evaluated scores of knowledge and practical abilities in clinical trials are shown in Table S2, Figs. 2 and 3. The total mean score for knowledge and practical abilities was 2.11 ± 0.91, and 75.76% of respondents got a mean score lower than 3. The total mean score for practical ability (2.02 ± 0.92) was lower than that for knowledge mastery (2.20 ± 0.93), *P* < 0.01 (Table S2). The order of the five dimensions of the total mean integrated knowledge and practical abilities score from highest to lowest were: subject recruitment and random grouping (2.30 ± 0.99), clinical trial protocol (2.25 ± 1.08), ethics in clinical trials (2.08 ± 1.00), data management and statistical analysis (1.99 ± 0.91), case report form (CRF) (1.98 ± 1.00) respectively (Table S2). For knowledge mastery, the dimension of CRF got the lowest score (2.03 ± 1.03); and for practical abilities, the dimension of data management and statistical analysis got the lowest score (1.87 ± 0.93). It is worth noting that the total mean score for practice was lower than that of knowledge in all five dimensions, and the differences were statistically significant in dimensions 2–5 (Fig. 2, Table S2).

- *Influence of students’ characteristics on the total score*
  There were no significant differences in the total scores for different gender, grade, education system, participation in relevant systematic training and attitude towards clinical trials. But scores of those who had participated in clinical research (*P* = 0.04) and self-study (*P* < 0.01) were higher (Table S1).

- *Knowledge of and participation in simulation-based education*
  57.58% of the respondents haven’t heard of scenario simulation teaching and only 3.03% (2/66) students responded that they had taken simulation-based courses, namely a comprehensive experiment related to basic medicine and diagnostics. The issue of low fidelity was raised by one of these two students. This indicated that more publicity and promotion of simulation courses are needed.

### Steps 3 and 4: Definition of goals and allocation of educational strategies

This curriculum aims to establish a systematical competency-based training system to provide students with a theoretical and practical basis for participating in various types of clinical research projects with their tutors in the future. The objectives of each simulation class are shown in Supplementary files 4 and 5.

The preliminary framework for the curriculum was proposed as follows:

- The curriculum has been divided into Clinical Research 1 (Design and implementation of clinical trials) and Clinical Research 2 (Design and implementation of observational studies), both courses are worth 2 credits, each containing a total of 16 classes (including 4 theoretical classes, 1 precursor class and 11 simulation classes), 3 credit hours per class, and arranged in the 5^th^ and 7^th^ semesters respectively.
- These two curricula are both case-based. Clinical Research 1 is based on a published clinical trial of remdesivir in adults with severe COVID-19 [32]. Clinical Research 2 is based on a cross-sectional study investigating hypertension in Wuhan, Hubei Province, China, which has also been broadened to include a cohort study. Taking Clinical Research 1 as an example, the simulation contents include protocol writing, review and approval of clinical trials, registration, implementation of randomization and drug blinding, and statistical analysis. During these processes, students will mainly engage in simulation as an investigator, as well as a variety of simulation role changes (including statisticians, sponsors, monitors, ethics committee), in order to get an overall understanding of the entire flow of a RCT. Because our goal is not only to familiarize students with the work of a certain role as a “screw”, but also give them an overview of the entire “assembly line”, thus promoting better cooperation and instilling confidence to carry out their own clinical research.
- Curriculum arrangement and resource list are summarized in Table 1. The capacity and faculty resources of our center to develop the new curriculum have been assessed by the Teaching Management Office of the School of Medicine of our university. Each simulation class will include a short didactic teaching session covering the important concepts (estimated as about 30 min) before the hands-on practice. Assignments are all group based, 6 to 8 students in each group, 6 groups in total. Students will take on different roles or divide the work up between them. Operation manuals with objectives, materials, task-based simulation content and required deliverables will be provided and given to students in advance. Simulation materials included literature, documents and templates for learning; simulated cases, simulated data, and simulation tools such as medicine bottle with simulated drugs, envelopes. Oral reports, record files or the records of the process made by faculty can usually be used for the formative evaluation of each simulation. The last part of the simulation is debriefing. The guided reflection questions are organized by the gather-analyze-summarize method.

**Table 1:** Curriculum arrangement and resource list

Simulation domain	Design and implementation of clinical research (clinical trials, observational studies)
Time arrangement	2 courses in 2 semesters for 2 types of clinical research
Course arrangement	4 theoretical classes + 12 simulation classes
Participants	Medical undergraduates
Participation unit	Group (6 ~ 8 person)
Faculty	One lead facilitator, one assistant per two groups, one course secretary
Faculty requirement	Specializing in the design and implementation of clinical research and trained in teaching simulation courses (Except the secretary)
Equipment	A room with a good internet network, round tables, projection, preferably computer-equipped
Simulation technology	Multimedia, verbal role playing
Teaching materials	Operation manuals for students, syllabus and scenario scripts for faculty; simulation cases, literature, templates, simulated data, software, tools (such as medicine bottle, envelopes)
Platform	Online class group for students; teaching support group for teachers

### Step 5: Pre-implementation assessment and adjustment

A total of 27 experts (expertise in clinical research methodology, clinical trials, evidence-based medicine, clinical specialties, scientific research management) were consulted, and the 8 of the included experts hold senior professional ranks, 13 hold deputy senior rank, 5 hold intermediate rank, and 1 expert holds a different title (provincial manager of a pharmaceutical research and development company).

The quantitative and non-quantitative results of the expert consultation are shown in Table S3 and Table S4 respectively (Supplementary file 3). There was no class with a percentage of disagreement (got score 1 or 2) more than 10%. The mean scores of all classes were more than 4. Among the 16 classes of Clinical Research 1, *Auxiliary Examination* got the lowest score 4.04 (1.17), and was also the only class with a CV of more than 20% (CV = 29.01%). 14.71% (4/27) of experts thought the class was out of line with the clinical research design and nonspecific. We accepted their suggestions and deleted this class. 11.11% (3/27) of experts thought the design and production of CRFs were important and should be introduced first, and one expert added the collection of source data should be emphasized in the class on *Filling of CRFs*. Therefore, we added the class of *Design of CRF* and supplemented Simulation Class 9 with *Source Data Collection and Filling of CRFs*. For other details proposed by experts, that were revised, supplemented, or remained unchanged after discussion: see Table S4.

The final curriculum overview is shown in Table 2. The simulation class design scenario scripts, including detailed overview, preparation, simulation, evaluation and debrief section of each simulation class, are shown in Supplementary file 4 and 5.

**Table 2:** Curriculum Overview of Clinical Research 1 and Clinical Research 2

**Class Type**	**Number**	**Clinical research 1** **(**Randomized Controlled Trials**)**	**Clinical research 2** **(**Cross-Sectional Studies**)**
Theoretical Classes	1	Clinical Research Overview	Cross-Sectional Study
	2	The Ethics of Clinical Research and the Management of Clinical Trial Data	Cohort Study and Case Control Study
	3	Common Statistical Methods in Clinical Research	Screening and Diagnostic Tests
	4	Clinical Research Design: A Flipped Classroom for Randomized Controlled Trials	Real World Study
	Precursor Class	Introduction to the Teaching Plan for the Simulation of Randomized Controlled Trials	Introduction to the Teaching Plan for the Simulation of Cross-Sectional Studies
Simulation Classes	1	Writing A Clinical Trial Protocol I	Writing A Precursor Class Cross-Sectional Study Protocol I
	2	Writing A Clinical Trial Protocol II	Writing A Cross-Sectional Study Protocol II
	3	Design of Case Report Form	Development of Implementation Manual
	4	Review and Approval of Clinical Trials	Project Kick-Off Meeting (Project Training)
	5	Registration of Clinical Trials	Field Investigation Workflow (Pilot Investigation, Field Investigation)
	6	Generation of Random Sequence	Data Management
	7	Drug Blinding	Statistical Analysis of Cross-Sectional Study I
	8	Subject Recruitment, Informed Consent, and Random Allocation	Statistical Analysis of Cross-Sectional Study II
	9	Source Data Collection and Filling of Case Report Forms	Curriculum Expansion: From Cross-Sectional to Longitudinal Study
	10	Management and Report of Adverse Events	Writing A Clinical Research Report
	11	Unblinding and Statistical Analysis	Oral Defense

## Discussion

Consistent with our general needs assessment, most undergraduates in our survey haven’t participated in clinical research or undertaken relevant systematic training in clinical trials, but their motivation to learn and deliver clinical research is strong. The mean self-evaluated scores for knowledge and practical abilities of clinical trials were low, and the practical ability score was lower than that for knowledge. Poor self-evaluated knowledge about clinical trials especially clinical trial execution among medical students was also reported in 2016 in Egypt, for these are not commonly integrated into medical school curricula [33].

According to the results of self-assessment in our survey, undergraduates had the least knowledge of CRF, and the worst practical abilities in data management and statistical analysis, especially in database establishment and management. This suggested that undergraduates may have had no access to these aspects of clinical trials/research in their previous training, or may have lacked opportunities for practice. Therefore, we intentionally covered the relevant contents in our new curriculum, including two simulation classes related to the design and filling in of CRF and four simulation classes related to data management and statistical analysis. Only by making clear the students’ strengths and short comings can we aim to target the teaching accurately. This also suggested that attention should be paid to these areas of content when designing relevant clinical research courses.

Our survey indicated that students do not naturally acquire knowledge and skills related to clinical trials with increasing seniority. Similarly, the Egyptian study found there was no statistically significant difference between undergraduate and graduate students in knowledge of clinical trials [33]. Participating in clinical research and active learning would benefit the acquisition of knowledge and skills in clinical research in our survey. The medical students who acted as volunteers to help perform clinical studies during the COVID‐19 pandemic similarly expressed that whilst they had previously had few insights into the practicalities of organizing and conducting clinical trials, the research experience has been invaluable to their professional development and thought opportunities for clinical research placements should be incorporated into medical education [34]. The necessity of effective integration of research skills into the medical curriculum from very early in undergraduate careers was recognized [35–37], but there are multiple barriers limiting opportunities for research activities within hospital wards and in the community [36, 37]. Thus, we have creatively designed the simulation-based clinical research course to restore the real clinical research scenes, meeting the needs of student participation; meanwhile the integrated problem-based learning teaching methods could stimulate students’ active learning.

The simulation-based medical education had been referred to in the report of the early twentieth century [38] and showed patient benefits [39]. Compared with extensive literature on the use of simulation in clinical skills teaching, there is limited literature reporting simulation in research.

Peacock and Thiel made early explorations of simulation in research teaching, and one was the famous cookie experiment [6, 13],but at that time, the simulation theory was not well developed. The description of high-fidelity simulation in clinical research training was first reported in 2004 [40]. The study used a human patient simulator as a simulated enrolled study patient during the training for study coordinators and found the simulation exercise could increase their confidence in developing a clinical research protocol. Brindley theoretically outlined potential benefits of the incorporation of simulation into clinical research, such as decreasing protocol learning-curve, improving study design and increasing participant safety [41].

For simulation teaching in specific research design, an outbreak investigation simulation was developed to teach nursing students principles of epidemiology in 2016 [42]. A Korean study reported the feasibility and good effects of a course including a simulated clinical crossover trial to teach clinical studies for undergraduates, during which students were randomly grouped to distinguish between two different types of cola as simulated subjects [14]. Another study in Australia described the use of a simulation workshop to teach the research processes to midwifery and nursing students, and also showed an increase in students’ interest in and knowledge of the topic [15]. However, these courses were not systematic enough for the limited lesson periods, only involved a single simulated type of research and had little student participation as simulated researchers, which is the main role in their future career. These limitations would restrict the complete simulation of design and implementation of clinical research.

We have summarized the possible advantages of simulation in clinical research education in Table 3. The curricula we have developed have the following advantages: firstly, innovatively introduce simulation into clinical research courses. Secondly, the curricula were developed based on the systematic modified model including expert consultation as pre-implementation assessment. Attention should be paid to raising the standards of experts to avoid the halo effect that may lead to bias in the collection of questionnaire results. Thirdly, the simulated clinical research types incorporate both clinical trials and observational research, and students can play a diversity of participating roles. However, currently there is not yet any post-implementation evaluation; subsequent verification and reports are needed to test the effect of the curriculum.

**Table 3:** Advantages and challenges of simulation in clinical research education

Advantages	Challenges
•More practice and engagement •More interactivity •Teamwork training •Case-based coherence •Stronger sense of substitution, strengthens professionalism •Student-centered active learning •Comprehensive ability exercise •Allow students to make mistakes	•Significant effort expenditure by students and teachers •Insufficient faculty specializing in both clinical research and simulation teaching •Dependency on well-designed cases/scrips •Requirements for certain environment and equipment •Not applicable for a large class

This new curriculum is user friendly and has the potential for global acceptance even in underdeveloped areas of the world. As Gaba summarized, simulation applications are diverse and can be categorized by 11 dimensions including the purpose of the simulation activity, the technology applicable or required for simulations [10]. In contrast to clinical simulation, the simulation of clinical research does not focus on the treatment of diseases or symptoms, so it doesn’t need expensive highly complex simulation equipment or virtual reality, it mainly depends on simple technology such as multimedia, verbal role playing, good script design, and teacher guidance, which are relatively accessible. We have shared the key simulation class design scenario scripts in the attachment in order to make it easier for schools around the world to replicate and build this new curriculum.

However, there are still challenges in implementing the simulation curriculum, which are summarized in Table 3. Firstly, both students and teachers need to invest a lot of time and energy. In order to ensure the faculty’s initiative in the new curriculum, we should actively create a good internal (a user-friendly communication platform, moral boosters) and external (resource and financial support) environment, and provide enough training opportunities to facilitate faculty development. The challenge of resources has been mentioned above. Regarding the need for a round table for each group (6–8 person), there should be no more than 10 groups in a class, which may limit the application of the new curriculum in large classes. Increased student numbers make it necessary to split classes creating more teacher demand.

The development of the new curriculum was an attempt at addressing this need at a university in Wuhan, where the COVID-19 broke out in the early stage. One of the teaching cases was also designed as a simulation of clinical trial of a COVID-19 drug. It is to be acknowledged that the Wuhan background may have boosted the interest and participation of local college students in the curriculum. The students surveyed were all from a medical college in Wuhan so the sample size and its representativeness were limited. Attention should be paid to the similarity of population and background when the results of the survey are extended. Although in view of the high global prevalence of COVID-19, college students everywhere may also pay more attention to the development of clinical research than before. This may therefore be the best time to promote the simulation curriculum for clinical research.

## Conclusions

Decreasing physician-scientist and absent capability-based clinical research training during medical school have prompted us to enhance and innovate clinical research teaching. We have successfully developed a simulation-based clinical curriculum using a modified 7-step model. This paper adds details of a curriculum development and teaching design of a simulation-based clinical research teaching to a very small body of published reports. This is also the first report of such a curriculum being developed in China, presenting the efforts of Chinese faculty in curriculum innovation. We believe that the advantages of simulation teaching will improve the effect of traditional clinical research teaching and help the training of physician-scientists. But there still needs to be more validation and discussion in the future to address the remaining challenges.

## Supplementary Information

**Additional file 1.** Questionnaire on medical students’ cognition of clinical trials and scenario simulation teaching.

**Additional file 2.** Expert Consultation Forms for the Syllabus for Clinical Research Courses.

**Additional file 3.** Supplementary Figures and Tables.

**Additional file 4.** Simulation Class Design Scenario Script of Clinical Research 1 (ARandomized Controlled Trial Simulation).

**Additional file 5.** Simulation Class Design Scenario Script of Clinical Research 2 (ACross-sectional Study Simulation)

**Additional file 6. **Data.

## Data Availability

All data generated or analysed during this study are included in this published article and supplementary files.

## Abbreviations

COVID-19: Coronavirus Disease 2019
NIH: National Institutes of Health
SD: Standard deviation
GCP: Good clinical practice
NMPA: National Medical Products Administration
CV: Coefficient of variation
CRF: Case report form
CR: Clinical research
ICC: Intraclass correlation coefficient

## References

[1] Glasziou PP, Sanders S, Hoffmann T (2020). Waste in covid-19 research. BMJ.

[2] Wyngaarden JB (1979). The clinical investigator as an endangered species. N Engl J Med.

[3] Teo AR. The development of clinical research training: past history and current trends in the United States. Acad Med. 2009;84(4):433–8. 10.1097/ACM.0b013e31819a81c9 19318772

[4] Bensken WP, Nath A, Heiss JD, Khan OI (2019). Future directions of training physician-scientists: reimagining and remeasuring the workforce. Acad Med.

[5] Wang C, Liu Q (2013). A turning point for clinical research in China?. Lancet.

[6] Peacock D (1981). A simulation exercise on scientific research for use in undergraduate teaching. J Geogr High Educ.

[7] Malik G, McKenna L, Griffiths D (2017). Using pedagogical approaches to influence evidence-based practice integration - processes and recommendations: findings from a grounded theory study. J Adv Nurs.

[8] Northway R, Parker M, James N, Davies L, Johnson K, Wilson S (2015). Research teaching in learning disability nursing: exploring the views of student and registered learning disability nurses. Nurse Educ Today.

[9] Bradley P (2006). The history of simulation in medical education and possible future directions. Med Educ.

[10] Gaba DM (2007). The future vision of simulation in health care. Qual Saf Health Care.

[11] Cook DA, Hatala R, Brydges R, Zendejas B, Szostek JH, Wang AT, Erwin PJ, Hamstra SJ (2011). Technology-enhanced simulation for health professions education: a systematic review and meta-analysis. JAMA.

[12] Schmidt E, Goldhaber-Fiebert SN, Ho LA, McDonald KM (2013). Simulation exercises as a patient safety strategy: a systematic review. Ann Intern Med.

[13] Thiel CA (1987). Views on research: the cookie experiment: a creative teaching strategy. Nurs Educ.

[14] Kim TH, Bae SJ, Kim DH, Kang JW (2020). Teaching clinical trials in Korean medicine: novel modules and student perceptions of importance and achievement. J Altern Complement Med.

[15] Lee N, Peacock A (2020). Using simulation to teach undergraduate nursing and midwifery students research design. Nurse Educ Pract.

[16] DE K, Thomas P, Kern D, Hughes M, Chen B (2016). A six-step approach to curriculum development. Curriculum development for medical education.

[17] Khamis NN, Satava RM, Alnassar SA, Kern DE (2016). A stepwise model for simulation-based curriculum development for clinical skills, a modification of the six-step approach. Surg Endosc.

[18] Zhang Q, Lee L, Gruppen LD, Ba D (2013). Medical education: changes and perspectives. Med Teach.

[19] Announcement of the National Health Commission on Issuing the Quality Management Practices for Drug Clinical Trials (No. 57 of 2020) (from National Medical Products Administration) [https://www.nmpa.gov.cn/yaopin/ypggtg/20200426162401243.html]

[20] Office of Clinical Research Training and Medical Education-NIH Clinical Center [https://www.cc.nih.gov/training/index.html]

[21] Boeynaems JM, Canivet C, Chan A, Clarke MJ, Cornu C, Daemen E, Demotes J, Nys KD, Hirst B, Hundt F (2013). A European approach to clinical investigator training. Front Pharmacol.

[22] CLIC : an online course for clinical investigators and their teams [https://clic.bio-med.ch/cms/Default]

[23] Chickering AW, Gamson ZF (1987). Seven principles for good practice in undergraduate education. The Wingspread J.

[24] INACSL Healthcare Simulation Standards of Best Practice. [https://www.inacsl.org/healthcare-simulation-standards-of-best-practice]

[25] Healthcare Simulation Dictionary –Second Edition [https://www.ahrq.gov/patient-safety/resources/simulation/terms.html]

[26] National League for Nursing. Simulation Innovation and Resource Center (SIRC).Simulation Design Template - Revised May 2019. [https://www.nln.org/education/education/sirc/sirc/sirc-resources/sirc-tools-and-tips#simtemplate]

[27] Working Committee for the Accreditation of Medical Education MoE, P. R. China: Standards for Basic Medical Education in China (2016 Revision). 2018. [http://wcame.bjmu.edu.cn/show.php?cid=20&id=158]

[28] Guiding Opinions on Comprehensively Promoting Health and Health Technology Innovation [http://www.gov.cn/xinwen/2016-10/12/content_5118171.htm]

[29] “Guiding Opinions on Accelerating the Innovation and Development of Medical Education” issued by the General Office of the State Council [http://www.gov.cn/zhengce/content/2020-09/23/content_5546373.htm]

[30] The department of clinical epidemiology and clinical trials was established in Capital Medical University [https://news.ccmu.edu.cn/syyw_12977/106279.htm]

[31] Zeng X, Li B, Lv J, Tian G (2017). Analysis of clinical research current status and its development trend (Chinese). Chin J Evid-Based Cardiovasc Med.

[32] Wang Y, Zhang D, Du G, Du R, Zhao J, Jin Y, Fu S, Gao L, Cheng Z, Lu Q (2020). Remdesivir in adults with severe COVID-19: a randomised, double-blind, placebo-controlled, multicentre trial. Lancet.

[33] Alfaar AS, Hassan WM, Bakry MS, Ezzat S (2017). Clinical research recession: training needs perception among medical students. J Cancer Educ.

[34] Prior SD, McKinnon T, Gresty V, Mulligan M, Richards L, Watson A, Green CA (2021). COVID-19: medical students in clinical research. Clin Teach.

[35] Murdoch-Eaton D, Drewery S, Elton S, Emmerson C, Marshall M, Smith JA, Stark P, Whittle S (2010). What do medical students understand by research and research skills? Identifying research opportunities within undergraduate projects. Med Teach.

[36] Laidlaw A, Aiton J, Struthers J, Guild S (2012). Developing research skills in medical students: AMEE guide no 6.9. Med Teach.

[37] Metcalfe D (2008). Involving medical students in research. J R Soc Med.

[38] McGaghie WC, Issenberg SB, Petrusa ER, Scalese RJ (2010). A critical review of simulation-based medical education research: 2003–2009. Med Educ.

[39] Zendejas B, Brydges R, Wang AT, Cook DA (2013). Patient outcomes in simulation-based medical education: a systematic review. J Gen Intern Med.

[40] Taekman JM, Hobbs G, Barber L, Phillips-Bute BG, Wright MC, Newman MF, Stafford-Smith M (2004). Preliminary report on the use of high-fidelity simulation in the training of study coordinators conducting a clinical research protocol. Anesth Analg.

[41] Brindley PG, Dunn WF (2009). Simulation for clinical research trials: a theoretical outline. J Crit Care.

[42] Okatch H, Sowicz TJ, Teng H, Pilling L, Harmon M, Brewer C, Buttenheim A (2016). Nursing students as epidemiologists: a simulation approach. Clin Simul Nurs.

