# Supplementary material for: Clinical research capability enhanced for medical undergraduates: an innovative simulation-based clinical research curriculum development
Source: BMC Med Educ. 2022 Jul 14;22:543. doi: 10.1186/s12909-022-03574-6 (PMC9281572; doi:10.1186/s12909-022-03574-6)
Supplement: Supplementary file 1 — Additional file 1. Questionnaire on medical students’ cognition of clinical trials and scenario simulation teaching. [file 12909_2022_3574_MOESM1_ESM.docx]

**Supplementary file 1**

**Questionnaire on medical students’ cognition of clinical trials and scenario simulation teaching**

In the spring of 2020, the spread of Coronavirus Disease 2019 (COVID-19) pushed various R&D institutions, hospitals, and enterprises to carry out quantitative drug clinical trials, aiming to find and develop safe and effective drugs. However, problems like design, sample collecting, and analyses in some clinical trials have made it difficult to objectively and accurately evaluate their effectiveness, thus emphasizing the significance and necessity of scientific, standardized, and accurate clinical trials. The success rate of research and development can only be increased by improving the quality and level of standardization, adhering to the principles of science and norms, and making full use of precious resources.

Medical students are crucial participants in and organizers of the future’s clinical trials. Understanding their level of cognition and practical ability can help to carry out targeted teaching courses and explore efficient teaching modes, in the hope of increasing their interest, cognition, attitude, and ability. The questionnaire on medical students’ cognition of clinical trials and scenario simulation teaching held by the Second Clinical College of Wuhan University (Zhongnan Hospital) includes 3 parts: (1) basic information; (2) knowledge of and practical ability in clinical trials; (3) cognition of scenario simulation teaching. Data desensitization will be applied to protect your personal information and the answers to this questionnaire do not affect your test scores, it will take you about 15 minutes. If you agree to participate in this survey, please click “agree” and fill it out honestly. Thanks for your support!

Do you agree to take this survey?

Agree

Disagree

**Part 1: Basic Information**

Student ID:

Gender:

Male

Female

Age (18-45 years old):

Your major:

Clinical medicine

Stomatology

Medical Image Science

Medical Laboratory Science

Public Health

Pharmacy

Basic Medical Science

Nursing

Other

What is your education system?

Five-year/Four-year program

5+3

Eight-year program

Master degree candidate

Doctor degree candidate

Other

What is your current year in the educational system (i.e. the third year in an eight-year program)?

Have you ever participated in clinical research (including clinical trials) before?

Yes

No

(If you answer yes in last question) What is your role in clinical research?

Subject

Researcher

Have you ever taken relevant systematic training in clinical trials (not regular courses, like conferences or training classes)?

Yes

No

Have you ever studied relevant knowledge of clinical trials on your own initiative?

Yes

No

Would you like to carry out clinical trials if it’s possible?

Yes

No

To what extent do you agree or disagree with the following statements that “the increase in clinical research capabilities can improve medical staff’s clinical practice abilities”?


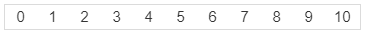


Strongly disagree (0) - Strongly agree (10)

To what extent do you agree or disagree with the following statements that “clinical trials can promote the development of medical science and thus benefit the patients”?


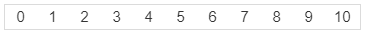


Strongly disagree (0) - Strongly agree (10)

**Part 2: Self-evaluation of knowledge and practical ability**

Note: The following self-evaluating items are aiming at assessing knowledge mastery and practical ability in clinical trials:

The evaluation score is respectively:

1= know absolutely nothing; 2=Don’t know; 3=Know something; 4=Know a reasonable amount; 5=Know everything.

Please fill the survey honestly reflecting your real situation, the results are only used for relevant designs of courses for the future.

1. Please evaluate your knowledge and practical ability about clinical trial protocols.

1-1 About the stipulated contents and writing standards of clinical trial protocol.

Knowledge mastery:


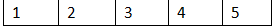


Practical ability:


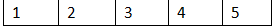


1-2 About the statistics of clinical trial protocol.

Knowledge mastery:


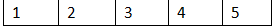


Practical ability:


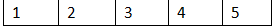


2. Please evaluate your knowledge and practical ability about the ethics in clinical trials.

2-1 As a researcher, how well do you know the submission process and material submitted to Medical Ethics Committee before starting a clinical trial?

Knowledge mastery:


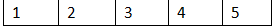


Practical ability:


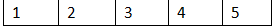


2-2 As a researcher, how well do you know the submission process and material submitted to Medical Ethics Committee during the clinical trial?

Knowledge mastery:


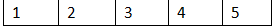


Practical ability:


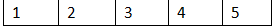


2-3 As a researcher, how much do you know about protecting subject’s rights and interests in clinical trials?

Knowledge mastery:


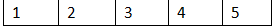


Practical ability:


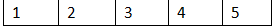


2-4 As a researcher, how well do you know about monitoring and reporting of adverse events in clinical trials?

Knowledge mastery:


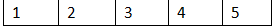


Practical ability:


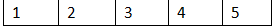


1. Please evaluate your knowledge and practical ability about the Case Report Form (CRF).

3-1 About designing a complete CRF

Knowledge mastery:


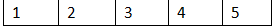


Practical ability:


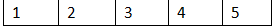


3-2 About the methods of transforming an original medical record to a CRF.

Knowledge mastery:


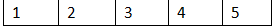


Practical ability:


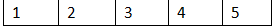


3-3 About the standard of filling, amending, and revising a CRF.

Knowledge mastery:


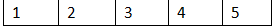


Practical ability:


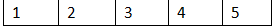


3-4 About transforming paper CRF to electronic ones.

Knowledge mastery:


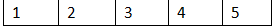


Practical ability:


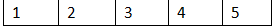


3-5 About the storage of paper and electronic CRF.

Knowledge mastery:


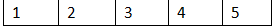


Practical ability:


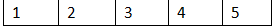


1. Please evaluate your knowledge and practical ability in subject recruitment and random grouping.

4-1 About how to use computers to randomize (use computer software to generate random sequences)

Knowledge mastery:


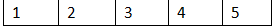


Practical ability:


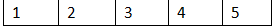


4-2 About how to achieve blinding in clinical trials.

Knowledge mastery:


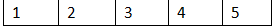


Practical ability:


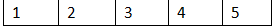


4-3 About how to maintain blinding during the research.

Knowledge mastery:


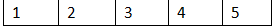


Practical ability:


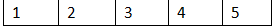


4-4 About how to recruit subjects for clinical trials.

Knowledge mastery:


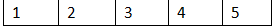


Practical ability:


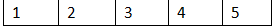


4-5 About how to communicate informed consent in clinical trials.

Knowledge mastery:


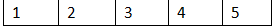


Practical ability:


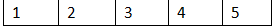


4-6 About how to screen subjects in clinical trials.

Knowledge mastery:


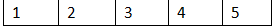


Practical ability:


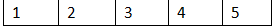


1. Please evaluate your knowledge and practical skills in data management and statistical analysis.

5-1 About the purpose and specific implementation steps of unblinding in clinical trials.

Knowledge mastery:


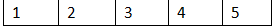


Practical ability:


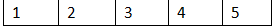


5-2 About formulating a complete statistical analysis plan (SAP) of a clinical trial.

Knowledge mastery:


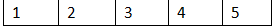


Practical ability:


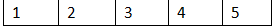


5-3 About the establishment and management of the clinical trial database.

Knowledge mastery:


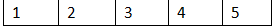


Practical ability:


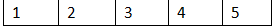


5-4 About data processing and statistical analysis of clinical trials.

Knowledge mastery:


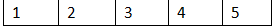


Practical ability:


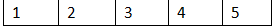


5-5 About interpreting of statistical analysis results in clinical trials.

Knowledge mastery:


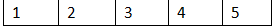


Practical ability:


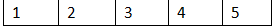


**Part 3 Scenario simulation teaching**

Notes: Scenario simulation teaching is a method to create an authentically designed scenario by teachers based on the teaching contents and objectives, where students can play a role and simulate a scenario to obtain knowledge and improve abilities. Please provide your current information on the courses with scenario simulation teaching you’ve completed.

1) Have you ever heard of scenario simulation teaching before?

Yes

No

2) Have any courses that you’ve taken used scenario simulation teaching before?

Yes

No

3) If you had, what’s the name of the course? ___________ (If you answer yes to Q2 please answer Q3-5)

4) If there are related courses, overall, do you support the scenario simulation teaching method?

Yes

No

5) (If you answer no to Q4) Please list your reasons if you don’t support it.

________
