# Supplementary material for: Clinical research capability enhanced for medical undergraduates: an innovative simulation-based clinical research curriculum development
Source: BMC Med Educ. 2022 Jul 14;22:543. doi: 10.1186/s12909-022-03574-6 (PMC9281572; doi:10.1186/s12909-022-03574-6)
Supplement: Supplementary file 2 — Additional file 2. Expert Consultation Forms for the Syllabus for Clinical Research Courses. [file 12909_2022_3574_MOESM2_ESM.docx]

**Supplementary file 2**

**Expert** **Consultation Forms for the Syllabus for Clinical Research Courses**

Dear Expert/Professor:

Thank you for sparing a time to provide valuable comments and suggestions for the syllabus for clinical research courses for eight-year program clinical medicine students.

The series of clinical research courses are for students majoring in clinical medicine (8-year-program). *Clinical Research 1* will be offered in the fifth semester (first term of Grade 3), and *Clinical Research 2* will be offered in the seventh semester (first term of Grade 5). Prior this course, students will have studied Medical Ethics, Medical Statistics, Epidemiology, SPSS statistical analysis and other courses (in Figure 1 for the detailed scientific research training flow chart). This course aims to establish a systematical and standardized scientific research training system to provide students with a theoretical and practical basis for participating in various types of clinical research projects with their tutors as soon as possible, so as to improve clinical research capabilities, increase students and tutors’ focus on clinical research and provide reference for management of the scientific research training process. The practical part of the clinical research courses will adopt simulation-based teaching. *Clinical Research 1* will simulate the whole process of the randomized controlled trial (RCT) of radixivir in the treatment of COVID-19. *Clinical Research 2* will simulate the process of a cross-sectional study on the prevalence and risk factors of hypertension in Wuhan, Hubei Province. We hoped that this teaching mode will bridge the gap between clinical epidemiology theory and practice, and clinical epidemiology courses will be gradually transformed from theory to practice through.

We hope you can proffer suggestions from the perspectives of teaching objectives, teaching strategies, teaching content and performance evaluations. Our decisions about implementation and follow up will be finalized after receiving and taking into account your comments.

If you have any questions about the contents in the consultation while filling out the form, you can communicate with us through the contact email or phone number below.

Sincerely thanks again!

Clinical Medicine major (8-year program) *Clinical Research 1*

and *Clinical Research 2* curriculum development working group

**Information for your reference before filling in the consultation form:**

Ref 1. The flow chart of current status about scientific research training for clinical medicine majors (8-year program) in our school；

Ref 2. Clinical Research Curriculum Overview.


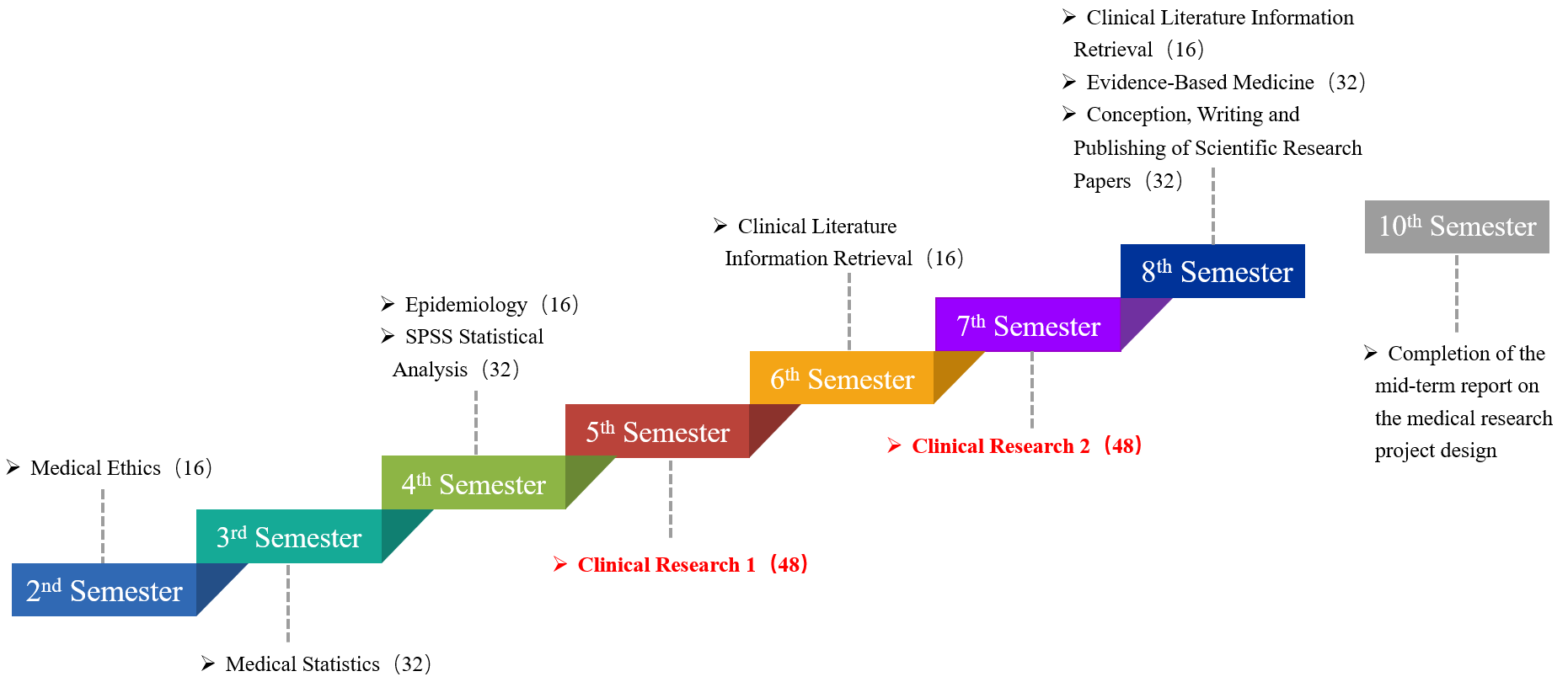


Ref 1. Flow Chart of Scientific Research Training for Clinical Medicine Majors (8-Year Program) In Our School (the numbers in parentheses are class hours)

Ref 2.1 Curriculum Overview of Clinical Research 1(16 Classes in Total)

| **Class Type** | **Number** | **Content** | **Duration**  **(Min)** |
| --- | --- | --- | --- |
| Theoretical Class | 1 | Clinical Research Overview | 3×45 |
|  | 2 | The Ethics of Clinical Research and the Management of Clinical Trial Data | 3×45 |
|  | 3 | Common Statistical Methods in Clinical Research | 3×45 |
|  | 4 | Clinical Research Design: a Flipped Classroom of Randomized Controlled Trials | 3×45 |
| Simulation Class | 5 | Precursor Class: Introduction to the Teaching Plan for Simulation of Randomized Controlled Trials | 3×45 |
|  | 6 | Writing a Clinical Trial Protocol I | 3×45 |
|  | 7 | Writing a Clinical Trial Protocol II | 3×45 |
|  | 8 | Review and Approval of Clinical Trials | 3×45 |
|  | 9 | Registration of Clinical Trials | 3×45 |
|  | 10 | Generation of Random Sequence | 3×45 |
|  | 11 | Drug Blinding | 3×45 |
|  | 12 | Subject Recruitment, Informed Consent, and Random Allocation | 3×45 |
|  | 13 | Auxiliary Examination (Laboratory Examination and Imaging Examination) | 3×45 |
|  | 14 | Filling of Case Report Forms | 3×45 |
|  | 15 | Management and Report of Adverse Events | 3×45 |
|  | 16 | Unblinding and Statistical Analysis | 3×45 |

Ref 2.2 Curriculum Overview of Clinical Research 2(16 Classes in Total)

| **Class Type** | **Number** | **Content** | **Duration**  **(Min)** |
| --- | --- | --- | --- |
| Theoretical class | 1 | Cross-Sectional Study | 3×45 |
|  | 2 | Cohort Study and Case Control Study | 3×45 |
|  | 3 | Screening and Diagnostic Test | 3×45 |
|  | 4 | Real World Study | 3×45 |
| Simulation class | 5 | Precursor Class: Introduction to the Teaching Plan for the Simulation of Cross-Sectional Studies | 3×45 |
|  | 6 | Writing a Cross-Sectional Study Protocol I | 3×45 |
|  | 7 | Writing a Cross-Sectional Study Protocol II | 3×45 |
|  | 8 | Development of Implementation Manual | 3×45 |
|  | 9 | Project Kick-Off Meeting (Project Training) | 3×45 |
|  | 10 | Field Investigation Workflow (Pilot Investigation, Field Investigation) | 3×45 |
|  | 11 | Data Management | 3×45 |
|  | 12 | Statistical Analysis of Cross-Sectional Study I | 3×45 |
|  | 13 | Statistical Analysis of Cross-Sectional Study II | 3×45 |
|  | 14 | Curriculum Expansion: From Cross-Sectional to Longitudinal Study | 3×45 |
|  | 15 | Writing A Clinical Research Report | 3×45 |
|  | 16 | Oral Defense | 3×45 |

Expert Consultation Form for the Syllabus of Clinical Medicine Major (8-year-program) in *Clinical Research 1*

Please give your opinion on the following contents of the syllabus for Clinical Research 1: overall teaching objectives, overall teaching strategies, teaching contents (listed in the order of class schedule, including the teaching objectives, teaching strategies and teaching contents of each class), performance evaluation, using five-point Likert scale：1= Strongly Disagree, 2= Disagree, 3= Uncertain, 4= Agree, 5= Strongly agree. Please tick "√" in the corresponding box. Also, if you disagree or have any further suggestions, please leave your valued comments in the corresponding position, thank you very much.

| **Proposed Content** | | **Agree or not**  **(1= Strongly Disagree, 2= Disagree, 3= Uncertain, 4= Agree, 5= Strongly agree)** | | | | |
| --- | --- | --- | --- | --- | --- | --- |
|  |  | **1** | **2** | **3** | **4** | **5** |
| **1 Overall Teaching Objectives** | | | | | | |
| The overall goal: to establish a systematic and standardized scientific research training system to provide students with a theoretical and practical basis for participating in various types of clinical scientific research projects with their tutors as soon as possible, so as to improve scientific research capabilities and standardize training standards. | |  |  |  |  |  |
| If you disagree or have any further suggestions, please leave your valued comments: | | | | | | |
| **2 Overall Teaching strategies** | | | | | | |
| Combination of theoretical classes and practical classes. | |  |  |  |  |  |
| Theoretical classes cover a flipped classroom with PBL (Problem-Based Learning, PBL) teaching. | |  |  |  |  |  |
| The practical classes are to carry out the simulation teaching of randomized controlled trials in groups (using published randomized controlled trials as examples). | |  |  |  |  |  |
| If you disagree or have any further suggestions, please leave your valued comments: | | | | | | |
| **3 Teaching contents** | | | | | | |
| **Theoretical Classes** | | | | | | |
| **3.1** **Clinical research overview** | |  |  |  |  |  |
| If you disagree or have any further suggestions, please leave your valued comments: | | | | | | |
| Teaching Objectives：   - Master: the basic principles and design of clinical research; how to choose a suitable research design based on the research question; - Familiarize: the ideas for topic selection in clinical research and how to construct clinical questions; how to implement clinical research; - Understand: possible bias and quality control of clinical research; relevant progress of clinical research. | |  |  |  |  |  |
| If you disagree or have any further suggestions, please leave your valued comments: | | | | | | |
| Teaching strategies: theoretical class | |  |  |  |  |  |
| If you disagree or have any further suggestions, please leave your valued comments: | | | | | | |
| Teaching contents：  The concept, classification, purpose, basic principles, design points, basic process, possible bias and quality control of clinical research; specifically include: how to extract scientific problems from clinical problems; how to extract research hypotheses from scientific assumptions; choose different types of research design according to clinical problems; how to implement clinical research, including database establishment and data collection; relevant progress in clinical research, such as large data and artificial intelligence. | |  |  |  |  |  |
| If you disagree or have any further suggestions, please leave your valued comments: | | | | | | |
| **3.2** The ethics of clinical research and the management of clinical trial data | |  |  |  |  |  |
| If you disagree or have any further suggestions, please leave your valued comments: | | | | | | |
| Teaching Objectives：   - Master: preparation of clinical research ethics application process and relevant materials, writing of ethics application; clinical research data management process and quality control methods. - Familiarize: the process and requirements of clinical research ethics management, the monitoring and reporting of adverse events, the general process and requirements of clinical research ethics review; the basic standards of clinical trial data collection, the concepts of GCP and SOP, and the quality control methods in the research process. - Understand: the establishment and responsibilities of the ethics committee, the requirements of research integrity; the definition and use of case report forms, and the importance of clinical trial data management quality control. | |  |  |  |  |  |
| If you disagree or have any further suggestions, please leave your valued comments: | | | | | | |
| Teaching strategies：  theoretical class(Theory and case teaching) | |  |  |  |  |  |
| If you disagree or have any further suggestions, please leave your valued comments: | | | | | | |
| Teaching contents：  The characteristics and ethical principles of scientific research; research integrity; ethical responsibilities of researchers; responsibilities and authority of ethics committees; clinical research ethics application process and preparation of relevant materials, ethical review process; purpose, significance, and responsibilities of clinical trial data management, Corresponding laws and regulations, data management procedures, etc. | |  |  |  |  |  |
| If you disagree or have any further suggestions, please leave your valued comments: | | | | | | |
| **3.3** **Common statistical methods in clinical research** | |  |  |  |  |  |
| If you disagree or have any further suggestions, please leave your valued comments: | | | | | | |
| Teaching Objectives：   - Master：the effect of medical statistics in clinical research, common statistical methods and applicable conditions； - Familiarize：common statistical indicators and interpretation, definition of confounding, adjustment methods for confounding factors； - Understand：statistical analysis plan and statistical analysis report. | |  |  |  |  |  |
| If you disagree or have any further suggestions, please leave your valued comments: | | | | | | |
| Teaching strategies：  theoretical class | |  |  |  |  |  |
| If you disagree or have any further suggestions, please leave your valued comments: | | | | | | |
| Teaching contents：  The relationship between clinical research and statistics, the basic concepts in medical statistics; the common methods and conditions of use in medical statistics, the influence of confounding factors and regression adjustment; the statistical considerations in randomized controlled trials. | |  |  |  |  |  |
| If you disagree or have any further suggestions, please leave your valued comments: | | | | | | |
| **3.4** **Clinical research design: a flipped classroom of randomized controlled trials** | |  |  |  |  |  |
| If you disagree or have any further suggestions, please leave your valued comments: | | | | | | |
| Teaching Objectives：  Overall: Exercise students' independent learning ability, critical thinking, communication and cooperation, and reporting skills.   - Master: the concept and significance of randomization, control, and blinding, the interpretation of research outcome indicators, and the methodology of critical reading of literature； - Familiarize: the advantages and disadvantages of randomized controlled experimental design； - Understand: the position of different research designs in the level of evidence in evidence-based medicine. | |  |  |  |  |  |
| If you disagree or have any further suggestions, please leave your valued comments: | | | | | | |
| Teaching strategies：  theoretical class(Problem-Based Learning，Flipped classroom, case teaching) | |  |  |  |  |  |
| If you disagree or have any further suggestions, please leave your valued comments: | | | | | | |
| Teaching contents：  Heuristic teaching, select the published randomized controlled trial used for simulation classes, and ask questions from the perspective of research design, research implementation, data analysis, result interpretation and reporting, and distribute case materials and question lists before class. Guide students to learn independently, actively explore and acquire knowledge; discuss in the classroom, collaborate to produce a group report, test the effect of students' autonomous learning, and foster the ability of students to collect and process information, actively explore innovation, and communicate and cooperate. Teachers reply Q&A. | |  |  |  |  |  |
| If you disagree or have any further suggestions, please leave your valued comments: | | | | | | |
| **Simulation Classes** | | | | | | |
| **3.5 Precursor class: Introduction to the Teaching Plan for the Simulation of Randomized Controlled Trials** | |  |  |  |  |  |
| If you disagree or have any further suggestions, please leave your valued comments: | | | | | | |
| Teaching Objectives：   - Familiarize：simulative teaching process and content; - Understand：the purpose, significance and method of simulation teaching; | |  |  |  |  |  |
| If you disagree or have any further suggestions, please leave your valued comments: | | | | | | |
| Teaching strategies：  theoretical class+ simulation class | |  |  |  |  |  |
| If you disagree or have any further suggestions, please leave your valued comments: | | | | | | |
| Teaching contents：  Teachers introduce the purpose, significance and content of simulated teaching; introduce test scoring standards; explain the criteria for evaluating the effectiveness of simulated teaching. Distribute practice manuals.  Students are grouped. Students discuss and ask questions based on the content of the teacher's lecture, and the teacher answers questions. | |  |  |  |  |  |
| If you disagree or have any further suggestions, please leave your valued comments: | | | | | | |
| **3.6 Writing A Clinical Trial Protocol I** | |  |  |  |  |  |
| If you disagree or have any further suggestions, please leave your valued comments: | | | | | | |
| Teaching Objectives：   - Master：importance of clinical trial protocol and key points for writing them； - Familiarize：stages, comparison types and design types of clinical trials； - Understand：revision and violation of clinical trial method | |  |  |  |  |  |
| If you disagree or have any further suggestions, please leave your valued comments: | | | | | | |
| Teaching strategies：  simulation class | |  |  |  |  |  |
| If you disagree or have any further suggestions, please leave your valued comments: | | | | | | |
| Teaching contents：  Teacher introduces the purpose and function of the research protocol and the standard items of the SPIRIT clinical trial protocol.  Students write a COVID-19 antiviral treatment research plan based on the template of the protocol. | |  |  |  |  |  |
| If you disagree or have any further suggestions, please leave your valued comments: | | | | | | |
| **3.7** **Writing A Clinical Trial Protocol II: Sample Size Calculation** | |  |  |  |  |  |
| If you disagree or have any further suggestions, please leave your valued comments: | | | | | | |
| Teaching Objectives：   - Master: the principle of replication in clinical trials, the importance of sample size estimation, and the factors affecting this; - Familiarize: principles of sample size estimation, sample size estimation of common designs; - Understand: the effect of different design types on the sample size estimation, the sample size is re-estimated. | |  |  |  |  |  |
| If you disagree or have any further suggestions, please leave your valued comments: | | | | | | |
| Teaching strategies：  simulation class | |  |  |  |  |  |
| If you disagree or have any further suggestions, please leave your valued comments: | | | | | | |
| Teaching contents：  The teacher introduces the steps of sample size estimation, and an example demonstrates the application of sample size estimation using the software.  Students use the COVID-19 antiviral treatment plan as an example to estimate the sample size and update the clinical research protocol. | |  |  |  |  |  |
| If you disagree or have any further suggestions, please leave your valued comments: | | | | | | |
| **3.8** **Review and Approval of Clinical Trials** | |  |  |  |  |  |
| If you disagree or have any further suggestions, please leave your valued comments: | | | | | | |
| Teaching Objectives：   - Master: preparation of clinical research ethics application process and relevant materials, writing of ethics application; - Familiarize: the general process and requirements of clinical trial ethics review, clinical trial review and approval; - Understand：establishment and responsibilities of ethics committees. | |  |  |  |  |  |
| If you disagree or have any further suggestions, please leave your valued comments: | | | | | | |
| Teaching strategies：  simulation class | |  |  |  |  |  |
| If you disagree or have any further suggestions, please leave your valued comments: | | | | | | |
| Teaching contents：  Teacher introduces the significance of ethical review and approval, the general process, the writing of ethical review applications, precautions and methods of recruiting subjects.  Students undertake role-plays in groups; teachers distribute ethical review submission materials for scientific research projects, and simulate ethical reviews (review and approval before the start of clinical trials, and review and approval during clinical trials). | |  |  |  |  |  |
| If you disagree or have any further suggestions, please leave your valued comments: | | | | | | |
| **3.9 Registration of Clinical Trials** | |  |  |  |  |  |
| If you disagree or have any further suggestions, please leave your valued comments: | | | | | | |
| Teaching Objectives：   - Master: progress of clinical trial registration, matters needing attention in registration information; - Familiarize: the importance of clinical research registration, the type of clinical research registered, the time of registration, common international and domestic clinical research registration databases, and general SCI journals' requirements for clinical research registration; - Understand: importance and significance of clinical research registration. | |  |  |  |  |  |
| If you disagree or have any further suggestions, please leave your valued comments: | | | | | | |
| Teaching strategies：  simulation class | |  |  |  |  |  |
| If you disagree or have any further suggestions, please leave your valued comments: | | | | | | |
| Teaching contents：  Teachers explain the importance of clinical research registration, the type of clinical research registered, the time of registration, international and domestic recognized clinical research registration databases, and general SCI journals' requirements for clinical research registration. Briefly introduce the registration process of American Clinical Trial and China Clinical Trial Registration Center, and the matters needing attention in registration information. The teacher summarizes the main items registered in the Chinese Clinical Trial Registration Center into the questionnaire star.  Students log in to the questionnaire in class to complete the relevant registration content. | |  |  |  |  |  |
| If you disagree or have any further suggestions, please leave your valued comments: | | | | | | |
| **3.10 Generation of Random Sequence** | |  |  |  |  |  |
| If you disagree or have any further suggestions, please leave your valued comments: | | | | | | |
| Teaching Objectives：   - Master: the generation of random sequence of simple randomization and the recording of blind code by the software; - Familiarize：common random allocation methods; - Understand：The significance of random allocation. | |  |  |  |  |  |
| If you disagree or have any further suggestions, please leave your valued comments: | | | | | | |
| Teaching strategies：  simulation class | |  |  |  |  |  |
| If you disagree or have any further suggestions, please leave your valued comments: | | | | | | |
| Teaching contents：  Teachers introduce concept of randomization, significance of random allocation and common methods and operating process of random allocation using the software.  Students play the role of third-party statisticians and use SPSS software to generate random sequence to randomly allocate 100 subjects into experimental group and control group at a 1:1 ratio. | |  |  |  |  |  |
| If you disagree or have any further suggestions, please leave your valued comments: | | | | | | |
| **3.11 Drug Blinding** | |  |  |  |  |  |
| If you disagree or have any further suggestions, please leave your valued comments: | | | | | | |
| Teaching Objectives：   - Master：the general process of drug blinding; - Familiarize：the technology of blinding implementation; - Understand：the significance of blind design. | |  |  |  |  |  |
| If you disagree or have any further suggestions, please leave your valued comments: | | | | | | |
| Teaching strategies：  simulation class | |  |  |  |  |  |
| If you disagree or have any further suggestions, please leave your valued comments: | | | | | | |
| Teaching contents：  Teachers introduce concept of blinding, significance of blinding, classification of blinding, technology of blinding implementation, concept of emergency blind disclosure  Students bring the blind code files generated in the last class and recall or reproduce the process of random sequence generation. Drug labels and emergency letters would be designed according to the randomized grouping scheme. | |  |  |  |  |  |
| If you disagree or have any further suggestions, please leave your valued comments: | | | | | | |
| **3.12 Subject Recruitment, Informed Consent, and Random Allocation** | |  |  |  |  |  |
| If you disagree or have any further suggestions, please leave your valued comments: | | | | | | |
| Teaching Objectives：   - Master: can give information about informed consent in accordance with GCP regulations; can randomly allocate qualified subjects and enter the group for medication. - Familiarize: Subject screening process and corresponding communication - Understand: the challenges, strategies and process of subject recruitment | |  |  |  |  |  |
| If you disagree or have any further suggestions, please leave your valued comments: | | | | | | |
| Teaching strategies：  simulation class | |  |  |  |  |  |
| If you disagree or have any further suggestions, please leave your valued comments: | | | | | | |
| Teaching contents：  Teachers introduce the background of subject recruitment  , requirements of informed consent in Good Clinical Practice (GCP), contents of informed consent, inclusion criteria, exclusion criteria, exit criteria, random allocation and recall of blinding.  Students work in groups to simulate participants and researchers, respectively, to formulate and simulate the participants recruitment plan; to drill the informed consent process and sign the informed consent form; to screen and record subjects based on simulated medical records; to be selected as qualified recipients who will accept the corresponding randomized drugs. | |  |  |  |  |  |
| If you disagree or have any further suggestions, please leave your valued comments: | | | | | | |
| **3.13** **Auxiliary Examination (Laboratory Examination and Imaging Examination)** | |  |  |  |  |  |
| If you disagree or have any further suggestions, please leave your valued comments: | | | | | | |
| Teaching Objectives：   - Master: assist in the writing of relevant content plans and relevant personnel training content; - Familiarize: the contents of auxiliary examination items in clinical research, auxiliary examination standard operating procedures, preparations and precautions before and after examination, care after examination and precautions for subjects, etc. | |  |  |  |  |  |
| If you disagree or have any further suggestions, please leave your valued comments: | | | | | | |
| Teaching strategies：  simulation class | |  |  |  |  |  |
| If you disagree or have any further suggestions, please leave your valued comments: | | | | | | |
| Teaching contents：  Teachers explain the contents of auxiliary examination items, auxiliary examination standard operating procedures, preparations and precautions before and after examination, care and precautions for subjects, etc.  Students are divided into groups to write related content plans for auxiliary examinations, simulate related personnel training content (doctors, nurses), and fill in the sample collection information card. | |  |  |  |  |  |
| If you disagree or have any further suggestions, please leave your valued comments: | | | | | | |
| **3.14 Filling of Case Report Forms** | |  |  |  |  |  |
| If you disagree or have any further suggestions, please leave your valued comments: | | | | | | |
| Teaching Objectives：   - Master: The contents of the case record book, and correctly fill in the medical history, clinical manifestations, laboratory and auxiliary examinations, primary and secondary and other outcome indicators on the case report form; - Familiarize: The location and function, content design, format, design process of the case report form, standardized collection and management of data | |  |  |  |  |  |
| If you disagree or have any further suggestions, please leave your valued comments: | | | | | | |
| Teaching strategies：  simulation class | |  |  |  |  |  |
| If you disagree or have any further suggestions, please leave your valued comments: | | | | | | |
| Teaching contents：  Teachers explained the composition and writing specifications of the medical record book, the location and function of the case report form, content design, format, and instructions for filling in the case report form.  The students role-play in groups, the teachers distribute the prepared case record book and the case report form, and simulate the filling in of the case report form, including the content of the case screening period (7～0 days) and the subsequent stage (13～28 days) . | |  |  |  |  |  |
| If you disagree or have any further suggestions, please leave your valued comments: | | | | | | |
| **3.15 Management and Report of Adverse Events** | |  |  |  |  |  |
| If you disagree or have any further suggestions, please leave your valued comments: | | | | | | |
| Teaching Objectives：   - Master：judgment, management and reporting of adverse events, adverse drug reactions, and serious adverse events; - Familiarize：discovery of adverse events, MedDRA coding of adverse events, and implementation of emergency unblinding; - Understand：significance of safety evaluation. | |  |  |  |  |  |
| If you disagree or have any further suggestions, please leave your valued comments: | | | | | | |
| Teaching strategies：  simulation class | |  |  |  |  |  |
| If you disagree or have any further suggestions, please leave your valued comments: | | | | | | |
| Teaching contents：  Teachers introduce the importance of safety evaluation, definitions of common terms, MedDRA codes, evaluation of adverse events, and reports of serious adverse events.  Students judge whether adverse events have occurred in a number of simulated cases, and if so, perform corresponding management and reporting of adverse events, including judging whether it is an adverse event, judging whether it is a serious adverse event, whether urgent unblinding is necessary, describe the adverse event record, Adverse event handling and reporting. | |  |  |  |  |  |
| If you disagree or have any further suggestions, please leave your valued comments: | | | | | | |
| **3.16 Unblinding and Statistical Analysis** | |  |  |  |  |  |
| If you disagree or have any further suggestions, please leave your valued comments: | | | | | | |
| Teaching Objectives：   - Master：the use of different statistical methods and their implementation methods in software. Significance of unblinding randomized controlled trials and correct interpretation of statistical analysis results; - Familiarize：the specific steps of unblinding and the selection points of statistical methods; - Understand：unblinding procedures, statistical analysis plan and statistical analysis report. The significance of unblinding and interpretation of statistical results. | |  |  |  |  |  |
| If you disagree or have any further suggestions, please leave your valued comments: | | | | | | |
| Teaching strategies：  simulation class | |  |  |  |  |  |
| If you disagree or have any further suggestions, please leave your valued comments: | | | | | | |
| Teaching contents：  The teacher introduces the significance of unblinding, the standard operating specifications and precautions for unblinding, the main points of the clinical trial statistical analysis plan and statistical analysis report, and uses a simulation database to demonstrate how to unblind and how to perform statistical analysis in SPSS software. Statistical methods include the description of patients' baseline characteristics (including the statistical description of continuous variables and categorical variables), and the analysis process of effectiveness and safety (using t-test or non-parametric test, chi-square test, etc.).  The student group conducts simulated unblinding and statistical analysis based on the teacher's explanation, reports the results of the statistical analysis, and interprets the results. | |  |  |  |  |  |
| If you disagree or have any further suggestions, please leave your valued comments: | | | | | | |
| **4 Performance evaluation** | | | | | | |
| Formative evaluation  Formative evaluation (60%) | Randomized controlled trial flipped classroom: students’ reporting situation and performance, including the analysis of the literature from the perspective of research design, research implementation, data analysis, result interpretation and reporting (score：5%) |  |  |  |  |  |
|  | Simulation Class 1) The writing of research proposal: students write a COVID-19 antiviral treatment research proposal based on the template of the proposal (the sample size calculation part excluded) (score：5%) |  |  |  |  |  |
|  | Simulation Class 2) Sample size calculation: Students use the COVID-19 antiviral treatment plan as an example to estimate the sample size and update the clinical research plan (score：5%) |  |  |  |  |  |
|  | Simulation Class 3) Review and approval of clinical trials: students role-play in groups, simulate ethical review, fill in and submit relevant documents for ethical review (score：5%) |  |  |  |  |  |
|  | Simulation Class 4) Clinical research registration: The teacher summarizes the main items registered in the Chinese Clinical Trial Registration Center into the questionnaire star, and the students log in to the questionnaire star to complete the relevant registration content (score：5%) |  |  |  |  |  |
|  | Simulation Class 5) Random sequence generation: Simulation will randomly assign 100 subjects to the experimental group and the control group in a 1:1 ratio, sort out the random grouping results, record and submit blind documents (score：5%) |  |  |  |  |  |
|  | Simulation Class 6) Drug blinding: simulating the on-site blinding process of drugs, completing the blinding of drugs, submitting the blinding records, the drugs that have been blinded and emergency letters (score：5%) |  |  |  |  |  |
|  | Simulation Class 7) Participant recruitment, informed consent and enrollment assignment: discussion of the subject recruitment plan, simulated informed consent communication and signing, and decision and record of whether the subject is selected based on the simulated medical record (score：5%) |  |  |  |  |  |
|  | Simulation Class 9) Filling in the case report form: the teacher distributes the case record book and the case report form prepared by the teaching and research section to simulate the filling of the case report form (score：5%) |  |  |  |  |  |
|  | Simulation Class 10) Management and reporting of adverse events: Determine whether adverse events have occurred in a given number of simulated cases, and evaluate, record and report them (score：5%) |  |  |  |  |  |
|  | Simulation Class 11) Unblinding and statistical analysis: use SPSS software to perform statistical analysis based on the simulative database, report the results of statistical analysis, and interpret the results (score：10%) |  |  |  |  |  |
| Summative evaluation (40%) | Test (score：40%) |  |  |  |  |  |
| If you disagree or have any further suggestions, please leave your valued comments: | | | | | | |
| In addition to the contents of the above form, If you disagree or have any further suggestions, please leave your valued comments: | | | | | | |

**(This is the end of the expert consultation form for the syllabus of *Clinical Research 1*. Thank you for filling in! Next is the expert consultation form for the syllabus of "Clinical Research 2")**

Expert Consultation Form for the Syllabus of Clinical Medicine Major (8-year-program) in *Clinical Research 2*

Please give your opinion on the following contents of the syllabus for Clinical Research 2: overall teaching objectives, overall teaching strategies, teaching contents (listed in the order of class schedule, including the teaching objectives, teaching strategies and teaching contents of each class), performance evaluation, using five-point Likert scale：1= Strongly Disagree, 2= Disagree, 3= Uncertain, 4= Agree, 5= Strongly agree. Please tick "√" in the corresponding box. Also, if you disagree or have any further suggestions, please leave your valued comments in the corresponding position, thank you very much.

| **Proposed Content** | | **Agree or not**  **(1= Strongly Disagree, 2= Disagree, 3= Uncertain, 4= Agree, 5= Strongly agree)** | | | | |
| --- | --- | --- | --- | --- | --- | --- |
|  |  | **1** | **2** | **3** | **4** | **5** |
| **1 Overall Teaching Objectives** | | | | | | |
| The overall goal：to establish a systematic and standardized scientific research training system to provide theoretical and practical basis for students to participate in various types of clinical scientific research projects with their supervisors as soon as possible, so as to improve their scientific research ability. | |  |  |  |  |  |
| If you disagree or have any further suggestions, please leave your valued comments | | | | | | |
| **2 Overall teaching strategies** | | | | | | |
| Combination of theoretical and practical classes. | |  |  |  |  |  |
| The practical classes are to carry out the simulation teaching of cross-sectional research in groups (taking the hypertension survey in Wuhan, Hubei Province as a case). | |  |  |  |  |  |
| If you disagree or have any further suggestions, please leave your valued comments | | | | | | |
| **3 Teaching contents** | | | | | | |
| **Theoretical Class** | | | | | | |
| **3.1 Cross-sectional study** | |  |  |  |  |  |
| If you disagree or have any further suggestions, please leave your valued comments | | | | | | |
| Teaching Objectives:   - Master：Principles, application, main content and advantages and disadvantages of cross-sectional study; - Familiarize：Sampling method and sample size estimation method of sample survey; - Understand：Methods for data analysis of cross-sectional studies. | |  |  |  |  |  |
| If you disagree or have any further suggestions, please leave your valued comments | | | | | | |
| Teaching strategies  Theoretical class | |  |  |  |  |  |
| If you disagree or have any further suggestions, please leave your valued comments | | | | | | |
| Teaching contents  Concepts and characteristics of cross-sectional study; Type and purpose of research; Design and implementation: Determining the purpose of the study, the objects of the study, the type and sampling method, the estimation of the sample size; Data collection and analysis; Common bias and its control; Advantages and disadvantages of cross-sectional studies. | |  |  |  |  |  |
| If you disagree or have any further suggestions, please leave your valued comments | | | | | | |
| **3.2 Cohort study and Case control study** | |  |  |  |  |  |
| If you disagree or have any further suggestions, please leave your valued comments | | | | | | |
| Teaching Objectives:   - Master: The definition, characteristics, uses and types of cohort studies, and the main content of the design；Definition, characteristics, uses and types of case-control studies, and the main content of the design；Comparison of advantages and disadvantages between cohort studies and case-control studies - Familiarize：The determination of factors and outcomes of cohort study, the selection of research objects, and the estimation method of sample size；The selection of case group and control group subjects and the estimation method of sample size in case-control study. - Understand: Methods of data analysis for cohort studies and case-control studies. | |  |  |  |  |  |
| If you disagree or have any further suggestions, please leave your valued comments. | | | | | | |
| Teaching strategies: Theoretical class | |  |  |  |  |  |
| If you disagree or have any further suggestions, please leave your valued comments | | | | | | |
| Teaching contents  Concept and principle of cohort study; Types and Uses; Design and implementation: selection of study methods, determination of exposure and outcome, selection of study subjects, estimation of sample size, follow-up, data collection; Data collation and analysis; Common bias and its control; Advantages and disadvantages of research.  Concept and principle of case-control study; Type and use; Design and implementation: research object selection, sample size estimation, data collection; Data collation and analysis; Common bias and its control; Advantages and disadvantages of research. | |  |  |  |  |  |
| If you disagree or have any further suggestions, please leave your valued comments. | | | | | | |
| **3.3 Screening and diagnostic tests** | |  |  |  |  |  |
| If you disagree or have any further suggestions, please leave your valued comments | | | | | | |
| Teaching Objectives:   - Master: Concepts, objectives, application principles and differences between diagnostic tests and screening tests; Diagnostic test and screening evaluation methods and evaluation indicators; Determine test judgment criteria. - Familiarize: The evaluation design of diagnostic tests and screening; Methods to improve test efficiency. - Understand: Bias and control methods for diagnostic tests and screening tests. | |  |  |  |  |  |
| If you disagree or have any further suggestions, please leave your valued comments. | | | | | | |
| Teaching strategies: Theoretical class | |  |  |  |  |  |
| If you disagree or have any further suggestions, please leave your valued comments | | | | | | |
| Teaching contents  The concept and purpose of diagnostic tests; The authenticity, reliability and clinical application value of the diagnostic test; Methods to improve the efficiency of diagnostic tests; Common bias in evaluation of diagnostic tests.  Concepts, objectives and types of screening; Principles of implementation, selection and ethics of screening; Evaluation of the effectiveness of screening. | |  |  |  |  |  |
| If you disagree or have any further suggestions, please leave your valued comments | | | | | | |
| **3.4 Real world study** | |  |  |  |  |  |
| If you disagree or have any further suggestions, please leave your valued comments | | | | | | |
| Teaching Objectives:   - Master: Definitions and uses of real-world study; The thinking and process of real world study; Common types of clinical research problem identification and study design in research. - Familiarize: Data management process and data quality control. - Understand: Real-world study controls for bias and confounding. | |  |  |  |  |  |
| If you disagree or have any further suggestions, please leave your valued comments | | | | | | |
| Teaching strategies: Theoretical class | |  |  |  |  |  |
| If you disagree or have any further suggestions, please leave your valued comments. | | | | | | |
| Teaching contents:  Background of real-world studies; Definition and relationships of real world data, real world evidence, and real world research; Types and characteristics of real-world research; Key points of real-world study implementation: clinical research question construction, data sources, sample size estimation, ethical evaluation, data management, data analysis, and quality control; Common biases and confounding and controls in real-world studies; Limitations of real-world studies. | |  |  |  |  |  |
| If you disagree or have any further suggestions, please leave your valued comments. | | | | | | |
| **Simulation class** | | | | | | |
| **3.5 Precursor Class: Introduction to the Teaching Plan for the Simulation of Cross-Sectional Studies** | |  |  |  |  |  |
| If you disagree or have any further suggestions, please leave your valued comments. | | | | | | |
| Teaching Objectives:   - Familiarize: The process and content of simulation teaching - Understand: The purpose, significance and method of simulation teaching. | |  |  |  |  |  |
| If you disagree or have any further suggestions, please leave your valued comments. | | | | | | |
| Teaching strategies: Theoretical class and Simulation class. | |  |  |  |  |  |
| If you disagree or have any further suggestions, please leave your valued comments. | | | | | | |
| Teaching contents:  Teachers explain the purpose, significance and content of simulation teaching; Introducing the examination scoring standard; Explain the criteria for evaluating the effect of simulation teaching. Distribution of practice manuals.  Students are divided into groups. They then discuss and ask questions according to the teacher's teaching content, and teachers answer questions. | |  |  |  |  |  |
| If you disagree or have any further suggestions, please leave your valued comments. | | | | | | |
| **3.6 Writing A Cross-Sectional Study Protocol I** | |  |  |  |  |  |
| If you disagree or have any further suggestions, please leave your valued comments. | | | | | | |
| Teaching Objectives:   - Master: Key points for writing a cross-sectional study plan - Familiarize: General principles for the formulation of a cross-sectional study plan and preparation before writing. | |  |  |  |  |  |
| If you disagree or have any further suggestions, please leave your valued comments. | | | | | | |
| Teaching strategies: Simulation class | |  |  |  |  |  |
| If you disagree or have any further suggestions, please leave your valued comments. | | | | | | |
| Teaching contents  The teacher will introduce the purpose and function of the research program, the general principles for the development of the research program, and the preparation before the writing of the research program.  Students write the plan of the hypertension survey in Wuhan, Hubei Province according to the scheme template (excluding sample size estimation and questionnaire design). | |  |  |  |  |  |
| If you disagree or have any further suggestions, please leave your valued comments. | | | | | | |
| **3.7 Writing A Cross-Sectional Study Protocol II** | |  |  |  |  |  |
| If you disagree or have any further suggestions, please leave your valued comments. | | | | | | |
| Teaching Objectives:   - Master: Sample size estimation methods and factors affecting sample size estimation in cross-sectional studies; Questionnaire design. - Familiarize: Determination of data collection content - Understand: Questionnaire content, reliability and validity evaluation. | |  |  |  |  |  |
| If you disagree or have any supplement, please leave your precious comments. | | | | | | |
| Teaching strategies:  Simulation class | |  |  |  |  |  |
| If you disagree or have any further suggestions, please leave your valued comments. | | | | | | |
| Teaching contents:  The teacher introduces the steps of sample size estimation and demonstrates the application of sample size estimation using software. Teacher examples introduce the principles and key points of questionnaire design, pre-investigation and evaluation of questionnaire. Taking the hypertension survey plan of Wuhan, Hubei Province as an example, the students practiced the sample size estimation, designed the questionnaire, and updated the research plan. | |  |  |  |  |  |
| If you disagree or have any further suggestions, please leave your valued comments. | | | | | | |
| **3.8 Development of Implementation Manual** | |  |  |  |  |  |
| If you disagree or have any further suggestions, please leave your valued comments. | | | | | | |
| Teaching Objectives:   - Master: Develop the basic methods and procedures from the practice manual according to the research problem and purpose. - Familiarize: The basic elements of the practice manual, such as survey members, survey organization structure, common problems that may occur on the site and emergency response methods, etc. - Understand: The purpose, significance and method of compiling practical operation manual. | |  |  |  |  |  |
| If you disagree or have any further suggestions, please leave your valued comments. | | | | | | |
| Teaching strategies: Simulation class | |  |  |  |  |  |
| If you disagree or have any supplement, please leave your precious comments. | | | | | | |
| Teaching contents:  According to the content of the project, the teacher explains the basic elements of the survey practice manual and the basic methods of formulating the manual. Students will be arranged in groups, each group member under the guidance of the teacher is to develop the practice manual draft.  Teachers comment, guide and improve the students work and analyze and compare it with the prepared "Practice and Operation Manual for the Investigation of the Current Situation of Hypertension in Wuhan, Hubei Province" acting as a guide.  The teacher evaluates the simulation teaching effect, the students discuss and ask questions related to the teacher's teaching content, and the teacher answers questions. | |  |  |  |  |  |
| If you disagree or have any further suggestions, please leave your valued comments | | | | | | |
| **3.9** **Project Kick-Off Meeting (Project Training)** | |  |  |  |  |  |
| If you disagree or have any further suggestions, please leave your valued comments. | | | | | | |
| Teaching Objectives:   - Master: Training process and matters needing attention for all kinds of personnel involved in field simulation projects. - Familiarize: Site investigation preparation, site implementation content and matters needing attention - Understand: The overall framework for project implementation. | |  |  |  |  |  |
| If you disagree or have any further suggestions, please leave your valued comments. | | | | | | |
| Teaching strategies  Simulation class | |  |  |  |  |  |
| If you disagree or have any further suggestions, please leave your valued comments. | | | | | | |
| Teaching contents:  The teacher reviews the knowledge points of the theory course, sorts out the general framework of the project, and concurrently explains the purpose, content and matters needing attention in the field survey. The knowledge points are connected in a series, so that students can have a better understanding of the whole process of project implementation. Further explain the purpose, content, process and matters needing attention of the project kick-off meeting before the start of the field investigation, as well as the content of the field training simulation teaching.  Students will be divided into groups to complete the training as various participants of the project through role simulation, and to test the training effect of various participants of the project through role exchange, so as to make early preparation for the follow-up field investigation.  The teacher evaluates the simulation teaching effect, the students discuss and ask questions related to the teacher's teaching content, and the teacher answers questions. | |  |  |  |  |  |
| If you disagree or have any further suggestions, please leave your valued comments. | | | | | | |
| **3.10 Field Investigation Workflow (Pilot Investigation, Field Investigation)** | |  |  |  |  |  |
| If you disagree or have any further suggestions, please leave your valued comments. | | | | | | |
| Teaching Objectives:   - Master: Site investigation preparation, site implementation content, process and matters needing attention. - Familiarize: Signing informed consent on site, quality control content and matters needing attention on site investigation. - Understand: Purpose, significance and method of pre-investigation and field investigation. | |  |  |  |  |  |
| If you disagree or have any further suggestions, please leave your valued comments. | | | | | | |
| Teaching strategies  Simulation class | |  |  |  |  |  |
| If you disagree or have any further suggestions, please leave your valued comments. | | | | | | |
| Teaching contents:  Teachers explain the purpose, content, process and matters needing attention during field investigation, as well as the content of field investigation simulation teaching.  The students are divided into groups and complete the required field investigation contents through role simulation according to the practice manual, and complete the field investigation registration form, informed consent form, questionnaire form, field investigation transfer form, survey result feedback form and other forms.  The teacher evaluates the simulation teaching effect, the students discuss and ask questions related to the teacher's teaching content, and the teacher answers questions. | |  |  |  |  |  |
| If you disagree or have any further suggestions, please leave your valued comments. | | | | | | |
| **3.11 Data Management** | |  |  |  |  |  |
| If you disagree or have any further suggestions, please leave your valued comments. | | | | | | |
| Teaching Objectives:   - Master：Method and content of data verification, writing and handling of data query. - Familiarize：Database establishment and data entry. - Understand：The significance and importance of data management. | |  |  |  |  |  |
| If you disagree or have any further suggestions, please leave your valued comments. | | | | | | |
| Teaching strategies:  Simulation class | |  |  |  |  |  |
| If you disagree or have any further suggestions, please leave your valued comments. | | | | | | |
| Teaching contents  The teacher introduces the definition and significance of data management, the establishment of a database, the method of data collection, the method and content of data verification, the confidentiality and security of data.  According to the hypertension status questionnaire (the first two pages) designed previously, students simulate the establishment and data entry using EpiData database after the teacher's demonstration; Write the data verification plan, check the simulated data and write the data challenge file, leave the mark according to the verification provided by the teacher and sort the data into a data format that can be analyzed. | |  |  |  |  |  |
| If you disagree or have any further suggestions, please leave your valued comments. | | | | | | |
| **3.12 Statistical analysis of cross-sectional study I** | |  |  |  |  |  |
| If you disagree or have any further suggestions, please leave your valued comments. | | | | | | |
| Teaching Objectives:   - Master：The conditions of use for different statistical methods and the correct interpretation of SPSS statistical analysis results. - Familiarize：SPSS analysis of cross-sectional data of the specific operation steps and the selection of statistical methods. - Understand：General principles of common statistical analysis methods for cross-sectional data. | |  |  |  |  |  |
| If you disagree or have any further suggestions, please leave your valued comments. | | | | | | |
| Teaching strategies:  Theoretical class and Simulation class | |  |  |  |  |  |
| If you disagree or have any further suggestions, please leave your valued comments. | | | | | | |
| Teaching contents:  Teachers will introduce the characteristics of cross-sectional research data, common statistical analysis methods, SPSS statistical analysis procedures, and interpretation of statistical analysis results.  Utilizing the previous cross-sectional research data, students simulate the establishment and data import of SPSS database under the teacher's demonstration; Description analysis and hypothesis testing are carried out on the simulated data, and interpretation of statistical analysis results is conducted according to the teacher's instructions. | |  |  |  |  |  |
| If you disagree or have any further suggestions, please leave your valued comments. | | | | | | |
| **3.13 Statistical analysis of cross-sectional study II** | |  |  |  |  |  |
| If you disagree or have any further suggestions, please leave your valued comments. | | | | | | |
| Teaching Objectives:   - Master: Descriptive analysis of cross-sectional studies (standardization of commonly used analysis indicators and rates) and hypothesis testing (correlation analysis, univariate comparative analysis, multivariate regression analysis) - Familiarize: Tripartite distribution of disease (population, time, and space), cross-sectional characteristics of data. - Understand: Objective, significance and method of cross-sectional simulation analysis. | |  |  |  |  |  |
| If you disagree or have any supplement, please leave your precious comments. | | | | | | |
| Teaching strategies:  Theoretical class and Simulation class | |  |  |  |  |  |
| If you disagree or have any further suggestions, please leave your valued comments. | | | | | | |
| Teaching contents:  Teachers explain the purpose, significance and content of simulation teaching, as well as test scoring standards; The cross-sectional data and basic analysis ideas of simulation are introduced.  Students are divided into groups, complete the required statistical analysis content according to the practice manual, and write the statistical analysis report.  The teacher evaluates the simulation teaching effect, the students discuss and ask questions related to the teacher's teaching content, and the teacher answers questions. | |  |  |  |  |  |
| If you disagree or have any further suggestions, please leave your valued comments. | | | | | | |
| **3.14 Curriculum Expansion: From Cross-Sectional to Longitudinal Study** | |  |  |  |  |  |
| If you disagree or have any further suggestions, please leave your valued comments. | | | | | | |
| Teaching Objectives:   - Master: The purpose, similarities and differences, advantages and disadvantages of cross-sectional and longitudinal studies; Follow-up in longitudinal studies; Effect indicators and common analysis methods in prospective studies. - Familiarize: The design type and outcome indicator type of longitudinal study. - Understand: Causal inference in longitudinal studies. | |  |  |  |  |  |
| If you disagree or have any further suggestions, please leave your valued comments. | | | | | | |
| Teaching strategies:  Theoretical class and Simulation class | |  |  |  |  |  |
| If you disagree or have any further suggestions, please leave your valued comments. | | | | | | |
| Teaching contents:  Teachers explain the purpose, significance and content of simulation teaching and test scoring standards; Introduce the simulated longitudinal research data and basic analysis ideas.  Students are divided into groups, complete the required statistical analysis content according to the practice manual, and write the statistical analysis report  The teacher evaluates the simulation teaching effect, the students discuss and ask questions related to the teacher's teaching content, and the teacher answers questions. | |  |  |  |  |  |
| If you disagree or have any further suggestions, please leave your valued comments. | | | | | | |
| **3.15 Writing A Clinical Research Report** | |  |  |  |  |  |
| If you disagree or have any further suggestions, please leave your valued comments. | | | | | | |
| Teaching Objectives:   - Master: Methods of writing cross-sectional research papers and summarizing the main points of the papers. - Familiarize: Reporting criteria for cross-sectional study. | |  |  |  |  |  |
| If you disagree or have any further suggestions, please leave your valued comments. | | | | | | |
| Teaching strategies：Simulation class | |  |  |  |  |  |
| If you disagree or have any further suggestions, please leave your valued comments. | | | | | | |
| Teaching contents：  Learn to write and report cross-sectional research papers under teacher's supervision.  Students write papers in accordance with the Strobe standard and the paper format of international well-known medical journals. Write a paper in small groups based on the example of the course. Each group will write one paper. | |  |  |  |  |  |
| If you disagree or have any further suggestions, please leave your valued comments. | | | | | | |
| **3.16 Oral Defense** | |  |  |  |  |  |
| If you disagree or have any further suggestions, please leave your valued comments. | | | | | | |
| Teaching Objectives:   - Master：Interpret and report research results. | |  |  |  |  |  |
| If you disagree or have any further suggestions, please leave your valued comments. | | | | | | |
| Teaching Methods:  Simulation class | |  |  |  |  |  |
| If you disagree or have any further suggestions, please leave your valued comments. | | | | | | |
| Teaching contents: According to the requirements, the paper will be reported in groups, and the teachers and/or students will ask questions and make comments. Paper report for each group is 15 minutes, and questions and comments time 5 minutes. | |  |  |  |  |  |
| If you disagree or have any further suggestions, please leave your valued comments. | | | | | | |
| **4 Performance evaluation** | | | | | | |
| Formative evaluation (60%) | Simulation Class 2 and 3: Write the research plan: Students write the hypertension survey plan of Wuhan, Hubei Province according to the plan template (score: 10%). |  |  |  |  |  |
|  | Simulation Class 4: Preparation of practice operation manual: students complete the practice operation manual according to the plan (score: 10%). |  |  |  |  |  |
|  | Simulation Class 6: Workflow of On-site Investigation (Pre-investigation and Investigation): Students fill in forms such as On-site Investigation Registration Form, Informed Consent Form, Questionnaires, On-site Investigation Transfer Form, Survey Result Feedback Form, etc. according to the investigation simulation process (Score: 10%). |  |  |  |  |  |
|  | Simulation Class 7 Data Management: Submit relevant QES, REC files or necessary CHK files for database establishment and input in groups; Data verification plan; Data query files; Verified data (score: 10%). |  |  |  |  |  |
|  | Simulation Class 9 Cross-sectional Research Statistical Analysis II: Submit the following documents in groups: Statistical analysis results; Interpretation of clinical significance of statistical analysis results (score: 10%). |  |  |  |  |  |
|  | Simulation Class 10 cross-sectional research expansion: in groups, submit the following documents: statistical analysis results; Interpretation of clinical significance of statistical analysis results (score: 10%). |  |  |  |  |  |
| Summative evaluation (40%) | Paper and Presentation (Score: 40%) |  |  |  |  |  |
| If you disagree or have any further suggestions, please leave your valued comments. | | | | | | |
| In addition to the contents of the above table, if you have any other comments or further suggestions, please add them here. | | | | | | |

(This is the end of all the contents of the expert consultation form. Thank you again for filling in! Thank you very much!)
