# Supplementary material for: Clinical research capability enhanced for medical undergraduates: an innovative simulation-based clinical research curriculum development
Source: BMC Med Educ. 2022 Jul 14;22:543. doi: 10.1186/s12909-022-03574-6 (PMC9281572; doi:10.1186/s12909-022-03574-6)
Supplement: Supplementary file 3 — Additional file 3. Supplementary Figures and Tables. [file 12909_2022_3574_MOESM3_ESM.docx]

**Supplementary file 3：Supplementary Figures and Tables**

Figure S1 Flow chart of scientific research training for eight-year program medical students in our school (the numbers in parentheses are class hours; the courses marked in red are the target courses to develop)

Table S1 Characteristics of surveyed students, their total scores for knowledge and practical ability in clinical trials and differences test

Table S2 The self-evaluated knowledge and practical ability scores for clinical trials and difference tests between them

Table S3 Quantitative results of expert consultation for the syllabus of clinical research courses

Table S4 Non-quantitative results of expert consultation for the syllabus of clinical research courses


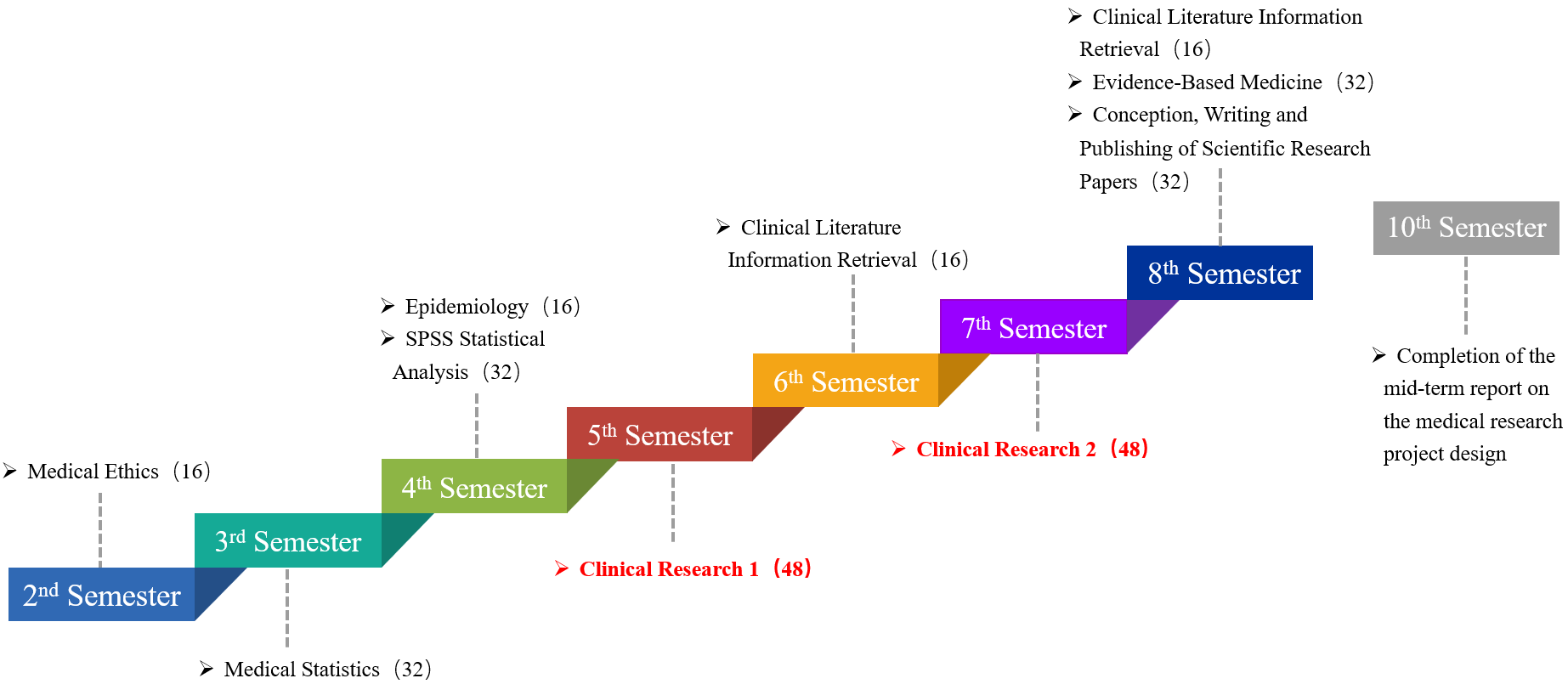
Figure S1 Flow chart of scientific research training for eight-year program medical students in our school（the numbers in parentheses are class hours; the courses marked in red are the target courses to develop）

Table S1 Characteristics of surveyed students, their total scores for knowledge and practical ability in clinical trials and differences test

| Characteristic | Item | n (%)/Mean (SD) | Total score^*^,  Median (Q1~Q3) | *Statistics^†^* | *P* |
| --- | --- | --- | --- | --- | --- |
| Gender | Male, n (%) | 34(51.52) | 91.00(64.00~132.00) | 0.78 | 0.44 |
|  | Female, n (%) | 32(48.48) | 85.50(48.00~117.50) |  |  |
| Age | Mean (SD) | 21.15(1.46) | - | - | - |
| Grade | 2, n (%) | 1(1.52) | 182.00(-) | 5.54 | 0.14 |
|  | 3, n (%) | 43(65.15) | 89.00(54.00~132.00) |  |  |
|  | 4, n (%) | 13(19.70) | 64.00(48.00~90.00) |  |  |
|  | 5, n (%) | 9(13.64) | 108.00(77.00~132.00) |  |  |
| Education system | Five-year/Four-year program (graduated as an undergraduate), n (%) | 20(30.30) | 80.00(47.00~126.00) | 2.79 | 0.25 |
|  | 5+3 program (graduated as a master), n (%) | 24(36.36) | 94.50(85.00~132.00) |  |  |
|  | Eight-year program (graduated as a MD), n (%) | 22(33.33) | 79.50(48.00~127.00) |  |  |
| Have you ever participated in clinical research (including clinical trials) before? | Yes, n (%) | 7(10.61) | 132.00(84.00~164.00) | 2.04 | **0.04** |
|  | No, n (%) | 59(89.39) | 87.00(50.00~110.00) |  |  |
| If you have participated in clinical research, what was your role in clinical research? | Subject, n (%) | 6(85.71) | 131.50(84.00~160.00) | 0.75 | 0.45 |
|  | Researcher, n (%) | 1(14.29) | 164.00(-) |  |  |
| Have you ever taken relevant systematic training in clinical trials (not regular courses, like conferences or training classes)? | Yes, n (%) | 10(15.15) | 102.50(77.00~160.00) | 1.64 | 0.10 |
|  | No, n (%) | 56(84.85) | 86.50(49.00~123.50) |  |  |
| Have you ever learned relevant knowledge about clinical trials on your initiative? | Yes, n (%) | 31(46.97) | 95.00(82.00~139.00) | 3.02 | **<0.01** |
|  | No, n (%) | 35(53.03) | 76.00(45.00~104.00) |  |  |
| Attitude to clinical trials |  |  |  |  |  |
| Would you like to carry out clinical trials if it’s possible? | Yes, n (%) | 62(93.94) | 88.50(50.00~132.00) | 0.43 | 0.68 |
|  | No, n (%) | 4(6.06) | 78.00(64.50~96.00) |  |  |
| Do you agree that “the increase in clinical research capabilities can improve medical staff’s clinical practice abilities”? | Mean (SD) | 8.47(1.53) | - | - | - |
| Do you agree that “clinical trials can promote the development of medical science and thus benefit the patients”? | Mean (SD) | 9.03(1.21) | - | - | - |

Abbreviations: SD, standard deviation; Q1, first quartile; Q3, third quartile; MD, Doctor of Medicine.

*: The total score is equal to the sum of the knowledge score and practice score of 22 questions in the questionnaire.

†: differences of the total score between two groups of respondents with different characteristics were tested by Wilcoxon signed rank test (*Statistics*=Z value), and differences among multiple groups (grade and education system) were tested by Kruskal Wallis test (*Statistics*=χ2 value)

Table S2 The self-evaluated knowledge and practical ability scores for clinical trials and difference tests between them

| Dimensions and questions | Knowledge mastery |  | Practical ability |  | Total |  | Difference test between knowledge mastery and practical ability* | |
| --- | --- | --- | --- | --- | --- | --- | --- | --- |
|  | Mean (SD) | <3 (%) | Mean (SD) | <3 (%) | Mean (SD) | <3 (%) | *S* | *P* |
| 1.Clinical trial protocol | 2.30(1.12) | 60.61 | 2.02(1.00) | 65.15 | 2.25(1.08) | 66.67 | 56.5 | 0.11 |
| The stipulated contents and writing standards of clinical trial protocol | 2.23(1.16) | 62.12 | 2.24(1.12) | 59.09 | 4.47(2.21) | - | -4.5 | >0.99 |
| The statistics of clinical trial protocol | 2.36(1.25) | 56.06 | 2.18(1.15) | 63.64 | 4.55(2.33) | - | 39.5 | **0.02** |
| 2. Ethics in clinical trials | 2.15(1.02) | 72.73 | 2.02(1.00) | 75.76 | 2.08(1.00) | 74.24 | 106.5 | **<0.01** |
| The submission process and material submitted to Medical Ethics Committee before starting a clinical trial | 2.09(1.15) | 66.67 | 1.95(1.06) | 69.70 | 4.05(2.16) | - | 27 | **0.03** |
| The submission process and material submitted to Medical Ethics Committee during the clinical trial | 1.94(1.05) | 71.21 | 1.85(1.00) | 74.24 | 3.79(2.02) | - | 7.5 | 0.06 |
| How to protect subject’s rights and interests in clinical trials | 2.35(1.17) | 54.55 | 2.20(1.14) | 60.61 | 4.55(2.26) | - | 23 | **0.02** |
| Monitoring and reporting of adverse events in clinical trials | 2.21(1.13) | 59.09 | 2.08(1.10) | 66.67 | 4.29(2.19) | - | 22.5 | **0.02** |
| 3. Case Report Form (CRF) | 2.03(1.03) | 71.21 | 1.93(0.99) | 74.24 | 1.98(1.00) | 72.73 | 58.5 | **<0.01** |
| Designing a complete CRF | 2.06(1.11) | 65.15 | 1.94(1.08) | 69.70 | 4.00(2.16) | - | 22 | **0.02** |
| The methods of transforming an original medical record to a CRF | 1.95(1.03) | 71.21 | 1.89(0.98) | 72.73 | 3.85(1.98) | - | 5 | 0.31 |
| The standard of filling, amending, and revising a CRF | 1.98(1.12) | 69.70 | 1.89(1.01) | 72.73 | 3.88(2.11) | - | 10.5 | **0.03** |
| Transforming a paper CRF to an electronic one | 2.00(1.07) | 68.18 | 1.92(1.10) | 69.70 | 3.92(2.14) | - | 12.5 | 0.18 |
| The storage of paper and electronic CRF | 2.17(1.16) | 60.61 | 2.00(1.08) | 71.21 | 4.17(2.17) | - | 23 | **0.02** |
| 4. Subject recruitment and random grouping | 2.44(1.02) | 62.12 | 2.16(1.02) | 71.21 | 2.30(0.99) | 69.70 | 171.5 | **<0.01** |
| How to use computers to randomize (use computer software to generate random sequences) | 2.18(1.16) | 60.61 | 2.00(1.10) | 69.70 | 4.18(2.19) | - | 18 | **0.01** |
| How to achieve blinding in clinical trials | 2.62(1.16) | 43.94 | 2.27(1.14) | 59.09 | 4.89(2.17) | - | 74.5 | **<0.01** |
| How to maintain blinding during the research | 2.41(1.19) | 51.52 | 2.14(1.12) | 63.64 | 4.55(2.21) | - | 47.5 | **<0.01** |
| How to recruit subjects for clinical trials | 2.26(1.14) | 57.58 | 2.06(1.15) | 65.15 | 4.32(2.23) | - | 34 | **0.01** |
| How to communicate informed consent in clinical trials | 2.68(1.18) | 42.42 | 2.36(1.21) | 54.55 | 5.05(2.29) | - | 77.5 | **<0.01** |
| How to screen subjects in clinical trials | 2.47(1.15) | 50.00 | 2.14(1.15) | 66.67 | 4.61(2.22) | - | 85.5 | **<0.01** |
| 5. Data management and statistical analysis | 2.11(0.94) | 72.73 | 1.87(0.93) | 80.30 | 1.99(0.91) | 78.79 | 159.5 | **<0.01** |
| The purpose and specific implementation steps of unblinding in clinical trials | 2.21(1.05) | 59.09 | 2.00(1.10) | 68.18 | 4.21(2.06) | - | 39.5 | **<0.01** |
| Formulating a complete SAP of a clinical trial | 2.09(1.06) | 68.18 | 1.88(0.94) | 71.21 | 3.97(1.96) | - | 52.5 | **<0.01** |
| The establishment and management of the clinical trial database | 1.86(0.97) | 75.76 | 1.74(0.97) | 77.27 | 3.61(1.90) | - | 18 | **0.04** |
| Data processing and statistical analysis of clinical trials | 2.15(1.08) | 63.64 | 1.85(1.00) | 74.24 | 4.00(2.00) | - | 68 | **<0.01** |
| Interpreting of statistical analysis results in clinical trials | 2.21(1.07) | 60.61 | 1.89(0.96) | 71.21 | 4.11(1.92) | - | 60 | **<0.01** |
| Total | 2.20(0.93) | 74.24 | 2.02(0.92) | 77.27 | 2.11(0.91) | 75.76 | 358.5 | **<0.01** |

Abbreviations: CRF= Case Report Form; SAP =statistical analysis plan;

Score is respectively: 1= Know absolutely nothing; 2=Don’t know; 3=Know something; 4=Know a reasonable amount; 5= Know everything.

Difference test between knowledge mastery and practical ability* was tested by signed rank test.

Table S3 Quantitative results of expert consultation for the syllabus of clinical research courses

| **Class Type** | **Number** | **Clinical research 1**  **(**Randomized Controlled Trials**)** | | | **Clinical research 2**  **(**Cross-Sectional Studies**)** | | |
| --- | --- | --- | --- | --- | --- | --- | --- |
|  |  | **Class Title** | **Mean (SD)** | **CV** | **Class Title** | **Mean (SD)** | **CV** |
| Theoretical Class | 1 | Clinical Research Overview | 4.71(0.46) | 9.77 | Cross-Sectional Study | 4.81(0.49) | 10.22 |
|  | 2 | The Ethics of Clinical Research and the Management of Clinical Trial Data | 4.78(0.51) | 10.60 | Cohort Study and Case Control Study | 4.62(0.7) | 15.11 |
|  | 3 | Common Statistical Methods in Clinical Research | 4.65(0.56) | 12.07 | Screening and Diagnostic Tests | 4.69(0.68) | 14.48 |
|  | 4 | Clinical Research Design: A Flipped Classroom of Randomized Controlled Trials | 4.76(0.66) | 13.94 | Real World Study | 4.62(0.9) | 19.45 |
| Simulation Class | Precursor Class | Introduction to the Teaching Plan for the Simulation of Randomized Controlled Trials | 4.62(0.64) | 13.81 | Introduction to the Teaching Plan for the Simulation of Cross-Sectional Studies | 4.85(0.37) | 7.59 |
|  | 1 | Writing A Clinical Trial Protocol I | 4.81(0.4) | 8.36 | Writing A Cross-Sectional Study Protocol I | 4.83(0.39) | 8.03 |
|  | 2 | Writing A Clinical Trial Protocol II | 4.96(0.2) | 4.12 | Writing A Cross-Sectional Study Protocol II | 4.92(0.28) | 5.63 |
|  | 3 | Review and Approval of Clinical Trials | 4.73(0.6) | 12.76 | Development of Implementation Manual | 4.58(0.76) | 16.55 |
|  | 4 | Registration of Clinical Trials | 4.81(0.4) | 8.36 | Project Kick-Off Meeting (Project Training) | 4.56(0.92) | 20.10 |
|  | 5 | Generation of Random Sequence | 4.75(0.74) | 15.52 | Field Investigation Workflow (Pilot Investigation, Field Investigation) | 4.8(0.50) | 10.42 |
|  | 6 | Drug Blinding | 4.81(0.8) | 16.66 | Data Management | 5(0.00) | 0.00 |
|  | 7 | Subject Recruitment, Informed Consent, and Random Allocation | 4.7(0.54) | 11.52 | Statistical analysis of cross-sectional study I | 4.81(0.49) | 10.22 |
|  | 8 | Auxiliary Examination (Laboratory Examination and Imaging Examination) | 4.04(1.17) | 29.01 | Statistical analysis of cross-sectional study II | 4.79(0.51) | 10.62 |
|  | 9 | Filling of Case Report Forms | 4.71(0.69) | 14.66 | Curriculum Expansion: From Cross-Sectional to Longitudinal Study | 4.71(0.91) | 19.28 |
|  | 10 | Management and Report of Adverse Events | 4.78(0.51) | 10.60 | Writing A Clinical Research Report | 4.81(0.49) | 10.22 |
|  | 11 | Unblinding and Statistical Analysis | 4.78(0.51) | 10.60 | Oral Defense | 4.83(0.48) | 9.96 |

Table S4 Non-quantitative results of expert consultation for the syllabus of clinical research courses

| Design/Class | Expert opinion overview | Number of experts who mentioned this opinion (%) | Response | Reason for Unchanged/ Specific solution |
| --- | --- | --- | --- | --- |
| Overall Teaching strategies | The order of courses should be observational research first, followed by RCTs. | 4(14.81) | Unchanged | RCT as gold standard for effectiveness research should be studied first. |
| Clinical Research 1 |  |  |  |  |
| Theoretical Class 2: The ethics of clinical research and the management of clinical trial data | Raise the profile of the purpose and necessity for application of clinical research ethics in the teaching objectives. | 1(3.70) | Accepted | - |
| Theoretical Class 3: Common statistical methods in clinical research | Add more practical contents. | 6(22.22) | Accepted | - |
| Theoretical Class 4: Clinical research design: a flipped course of randomized controlled trials | Add the introduction of the RCT report specification: CONSORT. | 2(7.41) | Accepted | - |
| Simulation Class 3：Review and approval of clinical trials | It was recommended participants observe the ethics approval meeting. | 1(3.70) | Unchanged | The ethics approval meeting does not usually allow observers.  If approved, we will consider adding it in later courses. |
| Simulation Class 4：Registration of clinical trials | Raise the importance level of the necessity and significance of clinical research registration in the teaching objectives. | 1(3.70) | Accepted | - |
| Simulation Class 5：Generation of random sequence | Add the content of allocation concealment. | 2(7.41) | Accepted | - |
|  | This part was too specialized and of little value to undergraduates. | 1(3.70) | Unchanged | We believed keeping this simulation will bring great benefit and increase students' understanding of randomization. |
|  | Add the content of random grouping method of unequal distribution | 1(3.70) | Accepted | - |
| Simulation Class 6: Drug Blinding | This part was too specialized and of little value to undergraduates. | 2(7.41) | Unchanged | We believed keeping this simulation will bring great benefit and increase students' understanding of randomization and blinding. |
| Simulation Class 8: Auxiliary Examination (Laboratory Examination and Imaging Examination) | It was out of line with the clinical research design and nonspecific, can be deleted. | 4(14.71) | Accepted | We have deleted this class and integrated it with Class 9 as *Source Data Collection and Filling of Case Report Forms*. |
| Simulation Class 9: Filling of case report forms | Design and production of case report forms should be introduced first. | 3(11.11) | Accepted | We have added a new class *Design of Case Report Form* after Simulation Class 2. |
|  | This part was out of line with the clinical research design and may be more suitable for CRC than clinicians. | 2(7.41) | Accepted | We have reduced the proportion of this part and integrated it with source data collection. |
|  | Emphasize the collection of source data. | 1(3.70) | Accepted | - |
| Performance evaluation | Two experts thought that the proportion of summative evaluations was low, but four other experts thought it was high. | 2(7.41), 4(14.71) | Unchanged | The overall proportion remains the same, but the difficulty of the summative evaluation test will be reduced as appropriate. |
|  | Distinguish the weight of formative evaluation of different practical classes according to the importance of the content and the amount of work done. | 2(7.41) | Accepted | - |
| Other comments | Add the contents of RCT report. | 3(11.11) | Accepted | We have included it in theory classes. |
|  | Emphasize the contents of GCP. | 2(7.41) | Accepted | We have included it in theory classes. |
| Clinical Research 2 |  |  |  |  |
| Overall Teaching contents | Add the contents of cohort studies. | 3(11.11) | Accepted | We have included it in the expansion of cross-sectional study. |
|  | Add the contents of case report studies and case series studies. | 2(7.41) | Unchanged | Limited by the course time, if there is more course time later, consider adding. |
| Theoretical Class 1: Cross-sectional study | Add the position and significance of cross-sectional studies in clinical research, and emphasize the clinical situations suitable for cross-sectional study design. | 3(11.11) | Accepted | - |
| Theoretical Class 2: Cohort study and case control study | Add special disease cohort cases, and emphasize the clinical situations suitable for these two different study designs. | 2(7.41) | Accepted | - |
| Theoretical Class 3: Screening and diagnostic tests | These two parts are actually more suitable for the practical class. | 1(3.70) | Unchanged | The simulation of Clinical Research 2 focused on the cross-sectional study and it is not appropriate to add screening and diagnostic tests. |
| Theoretical Class 4: Real world study | Recommendations were not to set up a separate class but to intersperse this content across various classes of research types. | 3(11.11) | Unchanged | Combined with the era of big data, the real world study is becoming more and more prominent and has its own characteristics. |
|  | Add the comparative teaching of real-world research and other research such as RCT. | 3(11.1) | Accepted | - |
| Simulation Class 3：Development of Implementation Manual | Delete this course content. | 1(3.70) | Unchanged | This class was a key part of quality control and should be kept. |
| Project Kick-Off Meeting | This class is not necessary. | 3(11.11) | Unchanged | Training all kinds of personnel in advance is a key part of quality control but is usually ignored in field surveys; therefore, this class should be kept. |
| Simulation Class 7：Statistical Analysis of Cross-Sectional Study I | There are limitations in software selection: the data analysis of large-scale cross-sectional research is relatively complex, and advanced statistical software such as Stata and R can be added to the course. | 1(3.70) | Unchanged | There is insufficient time within one class for students to master new programming software such as Stata and R. If time permits, the teacher will demonstrate how to use the advanced software. |
| Simulation Class 11：Oral Defense | Extend the time for question and comment. | 1(3.70) | Accepted | - |
| Performance evaluation | Two experts thought that the proportion of summative evaluations was low, while two experts thought it was high. | 2(7.41), 2(7.41) | Unchanged | - |
|  | Distinguish the weighting given to formative evaluation of different practical classes according to the importance of the content and the amount of work done. | 2(7.41) | Accepted | - |
| Other comments | It is suggested that public databases, such as SEER database, can be used for students to carry out data mining and related data analysis. | 1(3.70) | Unchanged | Public databases could be introduced in theory courses, but this would detract from the theme of this simulation. |
|  | Add a theoretical class in clinical research implementation project management and quality control | 1(3.70) | Accepted | We have interspersed the content in the theory courses. |

Abbreviations: RCT=Randomized Controlled Trial, GCP=Good Clinical Practice.
