# Supplementary material for: Clinical research capability enhanced for medical undergraduates: an innovative simulation-based clinical research curriculum development
Source: BMC Med Educ. 2022 Jul 14;22:543. doi: 10.1186/s12909-022-03574-6 (PMC9281572; doi:10.1186/s12909-022-03574-6)
Supplement: Supplementary file 4 — Additional file 4. Simulation Class Design Scenario Script of Clinical Research 1 (ARandomized Controlled Trial Simulation). [file 12909_2022_3574_MOESM4_ESM.docx]

Supplementary file 4

Simulation Class Design Scenario Script

A Randomized Controlled Trial Simulation

| **Field** | **Text** |
| --- | --- |
| Simulation Type | Short, didactic teaching and practical hands-on practice |
| Discipline | Clinical Medicine |
| Student Grade | Undergraduate |
| Simulation Research design | Randomized Controlled Trial (RCT) |
| Simulation Disease | Coronavirus Disease 2019 (COVID-19) |
| Simulation Hospital | Zhongnan Hospital or Leishenshan Hospital |
| Simulation Role /Mechanism | investigator (mainly), statistician, subjects, sponsor, monitor, independent ethics committee |
| Simulation Case | Remdesivir in adults with severe COVID-19: a randomized, double-blind, placebo-controlled, multicenter trial |
| Location | Discussion Classroom |
| Learning strategies | Group Learning |

Contents

| **Class Number** | **Class Content** | Duration  (min) |
| --- | --- | --- |
| 1 | Writing a Clinical Trial Protocol I | 3×45 |
| 2 | Writing a Clinical Trial Protocol II | 3×45 |
| 3 | Design of Case Report Form | 3×45 |
| 4 | Review and Approval of Clinical Trials | 3×45 |
| 5 | Registration of Clinical Trials | 3×45 |
| 6 | Generation of Random Sequence | 3×45 |
| 7 | Drug Blinding | 3×45 |
| 8 | Subject Recruitment, Informed Consent, and Random Allocation | 3×45 |
| 9 | Source Data Collection and Filling of Case Report Forms | 3×45 |
| 10 | Management and Report of Adverse Events | 3×45 |
| 11 | Unblinding and Statistical Analysis | 3×45 |

Class 1

Writing A Clinical Trial Protocol I

| **Overview tab** |  |
| --- | --- |
| Simulation Type | Short, didactic teaching and practical hands-on practice |
| Expected Theory Teaching Time | 30 minutes |
| Expected Simulation Run Time | 90 minutes |
| Guided Reflection Time | 15 minutes |
| Teacher’s Role | Instructors, facilitator |
| Student’s Simulation Role | Primary investigator |
| Simulated setting | Pharmaceutical factory |
| Learning Objectives | 1. Learn the importance of clinical study protocol and identify general items in a protocol.  2. Become familiar with: phases of clinical trial, comparative type and design type.  3. Understand: revision of clinical trial protocol, protocol deviation. |
| **Prepare tab** |  |
| Equipment List | Supplied by teachers   - PPT of theoretical knowledge - Clinical study protocol template - SPIRIT 2013 checklist - Standards for Quality Management of Drug Clinical Trials (China) - Evaluation of the Efficacy and Safety of Intravenous Remdesivir in Adult Patients with Severe Pneumonia caused by COVID-19 virus Infection: study protocol (English version)   Supplied by students   - Laptop |
| Theory Teaching  (30 mins) | - Purposes, functions and components of clinical study proposal - Introduce SPIRIT 2013 checklist - Introduce Standards for Quality Management of Drug Clinical Trials (China) - Exhibit 3 protocols for COVID-19 vaccine connected to tips in checklist and standards. |
| **Simulate tab** |  |
| Summary | Students read the COVID-19 protocol (English version), search for the key items in the protocol and write their own protocol (Chinese version). |
| Simulation operation (90 mins) | 1. Read the article “Evaluation of the Efficacy and Safety of Intravenous Remdesivir in Adult Patients with Severe Pneumonia caused by COVID-19 virus Infection: study protocol for a Phase 3 Randomized, Double-blind, Placebo controlled, multi-center trial”.  2. Fill out the protocol template, including:   - The background and purpose of the clinical trial. - Inclusion and exclusion criteria according to the purpose of the research, and determine the target population. - Research design type (center setting, randomization plan, blinding method, control group, comparison and design type). - Treatment and examination plan for included participants. - Efficacy evaluation (primary endpoint, secondary endpoint) and safety evaluation. - Sample size estimation (! Note: The content will be filled in next class). - Principle statistical analyses. - Quality control in the clinical trial. |
| **Evaluate tab** |  |
| Simulation Evaluation | The submitted protocol and roles of assignment in writing the protocol will be used for formative evaluation for each student.  **The scoring is based on the key points below:**   - Structure, completeness and logicality - Protocol summary - Role assignment |
| **Debrief tab** |  |
| Guided reflection questions  (15 mins) | These guided reflection questions are organized by the gather-analyze-summarize (GAS) method. The questions are presented to suggest topics that may inspire the debriefing conversation. Gather Information………………………………………………………….4 mins  - How did you feel throughout the simulation experience? - What were the difficulties you encountered during this simulation? - Would one of you describe this simulation procedure from your perspective?  Analyze …………………………………………………………………...8 mins  - Describe the importance and necessity of study protocol, the role of the protocol in a clinical study. - Describe the components of a study protocol. - How did you overcome the difficulties encountered during the simulation?  Summarize …………………………………………………………………3 mins  - What did you learn from this experience? - What would you like to do differently next time in a similar situation? |

Class 2

Writing A Clinical Trial Protocol II

| **Overview tab** |  |
| --- | --- |
| Simulation Type | Short, didactic teaching and practical hands-on practice |
| Expected Theory Teaching Time | 45 minutes |
| Expected Simulation Run Time | 75 minutes |
| Guided Reflection Time | 15 minutes |
| Teacher’s Role | Instructors, facilitator |
| Student’s Simulation Role | Statisticians, Sponsor |
| Simulated setting | Program office |
| Learning Objectives | 1. Grasp the importance and statistical considerations of sample size estimation  2. Become familiar with: key principles in clinical studies and power estimation  3. Understand: blinded and unblinded sample size re-estimation |
| **Prepare tab** |  |
| Equipment List | Supplied by teachers   - PPT of theoretical knowledge - Article “Statistical considerations of sample size determination in clinical trials” - G*Power 3.1 software - Evaluation of the Efficacy and Safety of Intravenous Remdesivir in Adult Patients with Severe Pneumonia caused by COVID-19 virus Infection: study protocol (English version) - Two examples for sample size estimation   Supplied by students   - Laptop |
| Theory Teaching  (45 mins) | - Why should we conduct sample size estimation? - Type I error and Type II error - Statistical difference vs Scientific difference - Key parameters in sample size estimation |
| **Simulate tab** |  |
| Summary | Based on the given parameters, students use available sample size estimation tool to calculate the optimal sample size required for the COVID-19 protocol, then describe essential information in a protocol. |
| Simulation operation (75 mins) | 1. Teachers answer questions proposed in theory teaching  2. Teachers summarize required key parameters  3. Download and Install G*Power 3.1. Introduce its menus and functions.  4. Demonstrate an example using a binary outcome  5. Demonstrate an example using a continuous outcome  6. Change key parameters and ask students to estimate their own sample size  7. Ask students to describe the results and complete the study protocol |
| **Evaluate tab** |  |
| Simulation Evaluation | The estimated sample size with specified parameters and description of sample size in the protocol are used for formative evaluation for each student.  **The scoring is based on the below key points:**   - Screenshot for sample size estimation and the same results - Appropriate description of sample size in the study protocol |
| **Debrief tab** |  |
| Guided reflection questions  (15 mins) | These guided reflection questions are organized by the gather-analyze-summarize (GAS) method. The questions are presented to suggest topics that may inspire the debriefing conversation. Gather Information………………………………………………………….3 mins  - How did you feel throughout the simulation experience? - What were the difficulties you encountered during this simulation? - Would one of you give a brief summary of this simulation?  Analyze …………………………………………………………………...8 mins  - Describe the importance and necessity of sample size estimation - Describe the required parameters in sample size estimation  Summarize …………………………………………………………………4 mins  - What did you learn from this experience? - What would you like to do differently next time in a similar situation? |

Class 3

Design of Case Report Form

| **Overview tab** |  |
| --- | --- |
| Simulation Type | Short, didactic teaching and practical hands-on practice |
| Expected Theory Teaching Time | 30 minutes |
| Expected Simulation Run Time | 90 minutes |
| Guided Reflection Time | 15 minutes |
| Teacher’s Role | Instructors, facilitator |
| Student’s Simulation Role | Investigator |
| Simulated setting | Program office |
| Learning Objectives | 1. Master the design principles and skills needed for case report forms;  2. Become familiar with the development of guidelines for filling in case report forms;  3. Understand the relationship between a case report form and trial protocol. |
| **Prepare tab** |  |
| Equipment List | Supplied by teachers   - PPT of theoretical knowledge - Clinical study protocol template - A number of paper copies (Remdesivir in adults with severe COVID-19: a randomized, double-blind, placebo-controlled, multicenter trial) - Case report form template: Case report form of a Phase II clinical study of famitinib malate combined with docetaxel in the treatment of advanced non-squamous and non-small cell lung cancer - Case report form and related literature: COVID-19 clinical trial case report forms and instructions for use   Supplied by students   - Laptop |
| Theory Teaching  (30 mins) | - The definition of a case report form - The design, production and approval management principles of case report forms - The relationship between case report forms and experimental schemes - The design principles and required skills for generating case report forms - The formulation of guidance for filling in case report form |
| **Simulate tab** |  |
| Summary | Design of case report form |
| Simulation operation (90 mins) | 1. After grouping, students should carefully read the case report form template (electronic version) and the COVID-19 clinical trial case report form and its instructions provided by the Teaching and Research Office, and familiarize themselves with the general contents of the case report form.  2. According to the template (electronic version) of the case report form prepared by the teacher, the students design the content of the case report form within the group. The content of the case report form shall include:   - Basic form filling instructions, clinical trial/time flow table; - Case screening period (-7~0 days)   Inclusion criteria, exclusion criteria, inclusion determination, informed consent, demographic information, comorbidities at admission, onset and admission, signs and symptoms at admission, laboratory tests, other tests, medications taken before admission and physical examination.   - Treatment period (1-10 days) and subsequent stages (14-28 days)   Laboratory tests (D3, D7 and D10), other tests, concomitant drug records, adverse events records and outcomes.  3. Questions encountered by each group during the design of case report form will be answered and discussed by four teaching assistants.  4. The teaching assistants summarize the problems encountered in the design process of the group, and the teacher will answer the questions. |
| **Evaluate tab** |  |
| Simulation Evaluation | The submitted electronic version of case report form is used for formative evaluation for each student.  **The scoring is based on the below key points:**   - Structure, completeness and logicality - Role assignment |
| **Debrief tab** |  |
| Guided reflection questions  (15 mins) | These guided reflection questions are organized by the gather-analyze-summarize (GAS) method. The questions are presented to suggest topics that may inspire the debriefing conversation. Gather Information………………………………………………………….3 mins  - How did you feel throughout the simulation experience? - What were the difficulties you encountered during this simulation? - Would one of you describe this simulation procedure from your perspective?  Analyze …………………………………………………………………...8 mins  - Describe the importance and necessity of design of case report forms, and the required design skills. - Describe the components of a case report form. - How did you overcome the difficulties encountered during the simulation?  Summarize …………………………………………………………………4 mins  - What did you learn from this experience? - What would you like to do differently next time in a similar situation? |

Class 4

Review and Approval of Clinical Research

| **Overview tab** |  |
| --- | --- |
| Simulation Type | Short, didactic teaching and practical hands-on practice |
| Expected Theory Teaching Time | 30 minutes |
| Expected Simulation Run Time | 90 minutes |
| Guided Reflection Time | 15 minutes |
| Teacher’s Role | Instructors, facilitator |
| Student’s Simulation Role | Investigator; Independent Ethics Committee member |
| Simulated setting | Independent Ethics Committee (IEC) |
| Learning Objectives | 1. Master the clinical trial ethics application process and related materials preparation, and ethical review application form writing.  2. Become familiar with the establishment and responsibilities of the independent ethics committee.  3. Understand the general procedures and requirements for clinical trial ethical review, review and approval of clinical trials. |
| **Prepare tab** |  |
| Equipment List | Supplied by teachers   - PPT of theoretical knowledge - List of submission materials for ethical review of scientific research projects - Letter of Ethical Submission - Application form for ethical review of biomedical research projects involving people - List of clinical trial research units and main participants - Investigator's resume - Template of clinical study protocol - Summary of revisions to the test protocol - Protocol deviation (PD) report form - Letter of ethical submission for protocol deviation report - Informed consent - Investigational brochure (IB) - Letter of ethics submission for safety incident report - Summary of serious adverse events - Subject recruitment notice - Comments on academic review and program discussion of the project   Supplied by students   - Laptop |
| Theory Teaching  (30 mins) | - The basic principles of ethics - The proposal of ethics principles - Ethics related norms - Principles of ethical review - Common ethical problems in clinical research - Common problems in ethical review - The whole process of ethical review - The general working procedures of independent ethics committee |
| **Simulate tab** |  |
| Summary | Students act as investigators and independent ethics committee members respectively to simulate review and approval of clinical research. |
| Simulation operation (75 mins) | 1. Preparation: Students read the attachments to understand the contents of clinical trial review and approval, and prepare for the subsequent ethical review and approval.  2. Review and approval before the commencement of clinical trials：   - Role division: Five students act as team members of IEC, and three other students act as researchers, one of whom is the primary researcher. IEC members should be familiar with working procedures. - For researchers: The 3 researchers in the group fill Ethical submission letter, application form for ethical review of biomedical research projects involving people, list of the clinical trial research units and main participating personnel, investigator's resume and subject recruitment notice. - For IEC members: they evaluate the clinical trial protocol written by the 3 researchers in Classes 1-2 and the informed consent made by the research group.   3. Review and approval of ongoing clinical trials:   - Simulate protocol deviation; - Simulate the management of serious adverse events. |
| **Evaluate tab** |  |
| Simulation Evaluation | NA |
| **Debrief tab** |  |
| Guided reflection questions  (15 mins) | These guided reflection questions are organized by the gather-analyze-summarize (GAS) method. The questions are presented to suggest topics that may inspire the debriefing conversation. Gather Information………………………………………………………….3 mins  - How did you feel throughout the simulation experience? - What were the difficulties you encountered during this simulation? - Would one of you describe this simulation procedure from your perspective?  Analyze …………………………………………………………………...8 mins  - Describe the importance and necessity of review and approval of clinical trials; - Describe the components in review and approval of clinical trials; - How did you overcome the difficulties encountered during the simulation?  Summarize …………………………………………………………………4 mins  - What did you learn from this experience? - What would you like to do differently next time in a similar situation? |

Class 5

Registration of Clinical Trials

| **Overview tab** |  |
| --- | --- |
| Simulation Type | Short, didactic teaching and practical hands-on practice |
| Expected Theory Teaching Time | 30 minutes |
| Expected Simulation Run Time | 90 minutes |
| Guided Reflection Time | 15 minutes |
| Teacher’s Role | Instructors, facilitator |
| Student’s Simulation Role | Investigator |
| Simulated setting | Website of Chinese Clinical Trial Registry |
| Learning Objectives | 1. Master the necessity and significance of clinical research registration; master the China Clinical Trial Registry registration process, and registration information notes.  2. Become familiar with the importance and types of clinical study registration, timing of registration, international and national clinical study registration databases. |
| **Prepare tab** |  |
| Equipment List | Supplied by teachers   - PPT of theoretical knowledge - Summarize the main items registered in Chinese Clinical Trial Registry to the online questionnaire before class - Reference contents registered by the author of the case paper on Clinical trial. gov: A Trial of Remdesivir in Adults With Severe COVID-19 - Registration contents of randomized, open, controlled clinical trials of darunavir or lopinavir/ritonavir tablets in combination with thymosin A1 in the Chinese Clinical Trial Registry for COVID-19   Supplied by students   - Laptop |
| Theory Teaching  (30 mins) | - Importance and definition of clinical trial registration - History and current status of clinical trial registration - Types of clinical trials registered - Timing of registration - Major clinical trial registration databases - Points for attention in registration information |
| **Simulate tab** |  |
| Summary | Students fill in the online questionnaire prepared by teachers to simulate the registration of clinical research on the website of Chinese Clinical Trial Registry. |
| Simulation operation (90 mins) | 1. After dividing into groups, students read the contents of the simulated reference paper registered by the author in Clinical Trial. gov: A Trial of Remdesivir in Adults with Severe COVID-19, and then read the contents of Randomized, open, controlled clinical trials of darunavir or lopinavir/ritonavir tablets in combination with thymosin A1 in the treatment of COVID-19 in the Chinese Clinical Trial Registry.  2. Teachers offer the online questionnaire used for simulation on registry through QR code in PPT.  3. After dividing into groups, the group leader should arrange the registration content for each group member.  Registered content should include the title, application for registration, contact details, basic information, primary researcher, research plan, informed consent, research purpose, drug ingredients or detail treatment, inclusion criteria, exclusion criteria, intervention, measurement location, recruitment, data collection and management.  4. The group will submit the questionnaire star after class as completing the registration. |
| **Evaluate tab** |  |
| Simulation Evaluation | The submitted questionnaire is used for formative evaluation for each student.  **The scoring is based on the below key points:**   - Structure, completeness and logicality - Role assignment |
| **Debrief tab** |  |
| Guided reflection questions  (15 mins) | These guided reflection questions are organized by the gather-analyze-summarize (GAS) method. The questions are presented to suggest topics that may inspire the debriefing conversation. Gather Information………………………………………………………….4 mins  - How did you feel throughout the simulation experience? - What were the difficulties you encountered during this simulation? - Would one of you describe this simulation procedure from your perspective?  Analyze …………………………………………………………………...8 mins  - Describe the importance and necessity of registration of Clinical Trials. - Describe the points for attention in registration information. - How did you overcome the difficulties encountered during the simulation?  Summarize …………………………………………………………………3 mins  - What did you learn from this experience? - What would you like to do differently next time in a similar situation? |

Class 6

Generation of Random Sequence

| **Overview tab** |  |
| --- | --- |
| Simulation Type | Short, didactic teaching and practical hands-on practice |
| Expected Theory Teaching Time | 30 minutes |
| Expected Simulation Run Time | 90 minutes |
| Guided Reflection Time | 15 minutes |
| Teacher’s Role | Instructors, facilitator |
| Student’s Simulation Role | Statistician |
| Simulated setting | Program office |
| Learning Objectives | 1. Create the generation of random sequence of simple randomization and record the blind code.  2. Become familiar with: common random allocation methods.  3. Understand: the significance of random allocation. |
| **Prepare tab** |  |
| Equipment List | Supplied by teachers   - PPT of theoretical knowledge - Sample files of blind code (electronic version)   Supplied by students   - Laptop with SPSS |
| Theory Teaching  (30 mins) | - Concept of randomization - Differentiation between random sampling and random allocation - Significance of random allocation - Common methods of random allocation, including simple randomization, block randomization, stratified randomization, central randomization - Operational process of random allocation - Allocation concealment |
| **Simulate tab** |  |
| Summary | Students play the role of third-party statisticians and use SPSS software to generate random sequence to allocate 100 subjects into experimental group and control group in a 1:1 ratio randomly. If time permits, a 2:1 ratio random allocation will also be simulated. |
| Simulation operation (90 mins) | 1.        Create subject number.  Create an empty SPSS data file and then create a variable from 1 to 100 as the subject number.  2.        Set random seed number.  In order to make the result of group randomization reproducible, it is necessary to set a random seed before grouping. For example, the date of group randomization (such as 20200724) can be used as the random seed.  3.      Generate random number.  Select a random function and generate a series of random numbers.  4.       Grouping according to the size of random numbers.  In this simulation, a total of 100 subjects would be randomly divided into two groups, with 50% in each group. Therefore, the median can be the cut-off point for group allocation.  5. Organize the results of group randomization into blind codes files according to the template files. |
| **Evaluate tab** |  |
| Simulation Evaluation | The blind code files that record the results of group randomization submitted in class by each student are used for formative evaluation.  **The scoring is based on the below key points:**   - Correct record of random allocation method - Correct record of random seed number - Correct random allocation |
| **Debrief tab** |  |
| Guided reflection questions  (15 mins) | These guided reflection questions are organized by the gather-analyze-summarize (GAS) method. The questions are presented to suggest topics that may inspire the debriefing conversation. Gather Information………………………………………………………….4 mins  - How did you feel throughout the simulation experience? - What were the difficulties you encountered during this simulation? - Would one of you describe this simulation procedure from your perspective?  Analyze …………………………………………………………………...8 mins  - Describe the definition of randomization. How is randomization achieved during the simulation? - Describe the contents of the blind code files. Why do we need to record this information? Can you reproduce the results from the previous time by doing it again? - How did you overcome the difficulties encountered during the simulation?  Summarize …………………………………………………………………3 mins  - What did you learn from this experience? - What would you like to do differently next time in a similar situation? |

Class 7

Drug Blinding

| **Overview tab** |  |
| --- | --- |
| Simulation Type | Short, didactic teaching and practical hands-on practice |
| Expected Theory Teaching Time | 30 minutes |
| Expected Simulation Run Time | 90 minutes |
| Guided Reflection Time | 15 minutes |
| Teacher’s Role | Instructors, facilitator |
| Student’s Simulation Role | Statisticians, sponsor |
| Simulated setting | Pharmaceutical factory |
| Learning Objectives | 1. Learn the general process of drug blinding.  2. Become familiar with: the technology of blinding implementation.  3. Understand: the significance of blind design. |
| **Prepare tab** |  |
| Equipment List | Supplied by teachers   - PPT of theoretical knowledge - Blind record file (paper version) - Medicine bottle with simulated drugs - Tag papers - Envelopes - Tape - Work area signs   Supplied by students   - Blind code files produced in the last class (paper version) - Pen |
| Theory Teaching  (30 mins) | - Concept of blinding - Significance of blinding - Classification of blinding - Technology of blinding implementation - Concept of emergency blind disclosure - Drug blinding process |
| **Simulate tab** |  |
| Summary | Students simulate the process of drug blinding, including designing drug labels and emergency letters according to the random grouping scheme, blinding according to the blinding process, finishing blinding record and handing over related items. |
| Simulation operation (90 mins) | 1.Prepare blind code.  Students bring the blind code files generated in the last class and recall or reproduce the process of random sequence generation.  2. Prepare for drug blinding.  Drug labels and emergency letters will be designed according to the randomized grouping scheme.   - Design labels: in clinical research, all packages of drugs, including the smallest package must be labeled and clearly defined as "clinical research medication". The label content should include some basic information about the drug, at least including: research title, drug number (random number). - Design emergency letter: The emergency letter is a sealed document designed to correspond with the drug number, which contains the specific grouping information of the drug number. The emergency letter and the drug number are one-to-one correspondence, and will be sent to the research center together with the blinded drugs.   3. Blinding and blinding records.  An independent statistician writes a draft of the blinding process and negotiates with the sponsor to determine the final process. In principle, the drug blinding process will follow the "blinding process". In general, it includes:   - Blind identification of drug: confirm that the drug group cannot be distinguished from the appearance of the outer packaging material of the test drug; - Check the packaging and storage of test drugs: confirm that the test drugs and control drugs are placed in their respective work areas; - Labeling: according to the blind code files, the label with number is pasted on the corresponding drugs and the outer packing box; - Drug transfer and Merger: transfer the drugs from work area B to work area A, merge them according to the number size, and load them into the large cartons distributed to each test center in sequence. - Seal and hand over the blind record: complete the blind record form (time, place, participants, process and signature of person in charge), sign and hand over the blind record form, drugs and emergency letters. |
| **Evaluate tab** |  |
| Simulation Evaluation | Take the team as a unit to check the completion of the following contents, which are used for formative evaluation:   - Blinded drugs and emergency letters; - Drug blinding record file.   **The scoring is based on the below key points:**   - Rational design of labels and emergency letters - Correct drug number corresponding to the blind code files - Orderly work arrangement |
| **Debrief tab** |  |
| Guided reflection questions  (15 mins) | These guided reflection questions are organized by the gather-analyze-summarize (GAS) method. The questions are presented to suggest topics that may inspire the debriefing conversation. Gather Information………………………………………………………….3 mins  - How did you feel throughout the simulation experience? - What were the difficulties you encountered during this simulation? - Would one of you give a brief summary of this simulation?  Analyze …………………………………………………………………...8 mins  - Describe the definition of blinding. How is blinding achieved during the simulation? - Describe the technology of blinding implementation and say what you used in this simulation. - Discuss the knowledge guiding your thinking surrounding these main difficulties. - How did you cooperate within the group?  Summarize …………………………………………………………………4 mins  - What did you learn from this experience? - What would you like to do differently next time in a similar situation? |

Class 8

Subject Recruitment, Informed Consent, and Random Allocation

| **Overview tab** |  |
| --- | --- |
| Simulation Type | Short, didactic teaching and practical hands-on practice |
| Expected Theory Teaching Time | 30 minutes |
| Expected Simulation Run Time | 90 minutes |
| Guided Reflection Time | 15 minutes |
| Teacher’s Role | Instructors, facilitator |
| Student’s Simulation Role | Investigator, subjects |
| Simulated setting | Hospital |
| Learning Objectives | 1. Know the necessity of informed consent, and implement informed consent and random allocation.  2. Become familiar with: the criteria of inclusion and exclusion of subjects, and the content of informed consent.  3. Understand: the challenges, strategies and process of subject recruitment. |
| **Prepare tab** |  |
| Equipment List | Supplied by teachers   - PPT of theoretical knowledge - Informed consent template - Medical records with hidden personal information - Subject screening record form   Supplied by students   - Blind code files sealed in envelopes (generated in the last class) - Blinded drugs (medicine bottles prepared in the last class) - Pen |
| Theory Teaching  (30 mins) | - Background of subject recruitment - Requirements for informed consent in Good Clinical Practice (GCP) - Contents of informed consent - Inclusion criteria, exclusion criteria, exit criteria - Random allocation - Drug management |
| **Simulate tab** |  |
| **Scenario 1** | **Subject Recruitment** |
| Teacher’s Role | Facilitator |
| Student’s Simulation Role | Investigator |
| Simulation operation (20mins) | 1.Discuss  In a group, discuss how to recruit subjects.  The following contents can be used for reference：   - Identify the person in charge of recruitment - Formulate recruitment plan, including recruitment area, recruitment method and strategy - Precautions for the implementation of recruitment work - Quality control and management   2.Share  After the discussion, some groups of students will be selected to share their work. |
| **Scenario 2** | **Informed consent** |
| Teacher’s Role | Facilitator |
| Student’s Simulation Role | Investigator, Subject, Witness, Guardian |
| Simulation operation (20mins) | Taking the group as the unit, the subject, investigator and witness are simulated respectively. According to the requirements of informed consent in GCP, simulate the informed consent process.  One or two of the following three situations are selected for simulation：   - One subject who can read and understand normally and one corresponding investigator - One subject who lacks reading ability, one corresponding witness and one investigator - One subject aged 10, one corresponding guardian and one investigator |
| **Scenario 3** | **Subject inclusion and drug allocation** |
| Teacher’s Role | Facilitator |
| Student’s Simulation Role | Investigator, Subject |
| Simulation operation (20mins) | 1. Screening of subjects   - Become familiar with the inclusion / exclusion criteria in the protocol - The group should function as a unit, using the medical records provided, judge whether the corresponding subjects can be included in the trial and simulate the conversation with subjects according to the results - Finish subject screening record form   2. Random allocation and drug assignment   - Check before random allocation (signature of informed consent, check of inclusion / exclusion criteria) - Drug assignment for eligible subjects. Each time a qualified subject is selected, drugs with corresponding number package are assigned to the subject according to the order of enrollment (the drugs have been pre blinded) |
| **Evaluate tab** |  |
| Simulation Evaluation | Take the group as a unit to check the completion of the following contents, which are used for formative evaluation:   - The simulation of informed consent communication; - The record of informed consent communication; - The judgment and record of whether the subject is selected or not.   **The scoring is based on the key points below:**   - Understandable, complete and honest information during informed consent communication - Correct judgment and record of subject selection |
| **Debrief tab** |  |
| Guided reflection questions  (15 mins) | These guided reflection questions are organized by the gather-analyze-summarize (GAS) method. The questions are presented to suggest topics that may inspire the debriefing conversation. Gather Information………………………………………………………….4 mins  - How did you feel throughout the simulation experience? - What were the difficulties you encountered during this simulation? - Would one of you give a brief summary of this simulation?  Analyze …………………………………………………………………....8 mins  - Describe the challenges of subject recruitment. How would you deal with these challenges? - Describe the comments and anxieties of subjects during informed consent communication. How could you better explain these problems to them from your perspective? - Describe the definition of randomization and blinding, and analyze how they were implemented during drug assignment.  Summarize …………………………………………………………………3 mins  - What did you learn from this experience? - What would you like to do differently next time in a similar situation? |

Class 9

Source Data Collection and Filling of Case Report Forms

| **Overview tab** |  |
| --- | --- |
| Simulation Type | Short, didactic teaching and practical hands-on practice |
| Expected Theory Teaching Time | 30 minutes |
| Expected Simulation Run Time | 90 minutes |
| Guided Reflection Time | 15 minutes |
| Teacher’s Role | Instructors, facilitator |
| Student’s Simulation Role | Investigator, Clinical Research Coordinator (CRC) |
| Simulated setting | Hospital |
| Learning Objectives | 1. Master the contents of the case book, and correct write a history, clinical manifestations, laboratory and auxiliary tests, primary, secondary and other outcome indicators on the case report form.  2.Become familiar with the position and function, content design, format and design process of case report forms.  3.Understand the definition and related concepts of source data, methods of source data collection, requirements for source data collection, preparation of documents related to source data collection, and data collection/entry process. |
| **Prepare tab** |  |
| Equipment List | Supplied by teachers   - PPT of theoretical knowledge - Several copies of paper (Remdesivir in adults with severe COVID-19: Randomised, double-blind, placebo-controlled, multicentre trial). - Several copies of medical records of COVID-19 patients (removing protected health information) - Case Report Form prepared by the Teaching and Research Office (provided by the teacher in class for the students). - Case Report Form (abbreviated edition) for students to fill in.   Supplied by students   - Laptop |
| Theory Teaching  (30 mins) | About source data   - The definition and related concepts of source data - Types of source data - Methods of source data collection - Requirements for source data collection - Data collection/input process - The process from source data generation to analysis data set generation   About filling of case report forms   - The composition and writing standards of the medical record - The position and function of the case report form - The association between source data, medical record and case report form - The instructions for filling in case report forms |
| **Simulate tab** |  |
| Summary | Three simulated cases are given to determine whether adverse events have occurred, and if yes, the corresponding management and reporting of adverse events will be carried out. |
| Simulation operation (90 mins) | 1. Teachers show source data with PPT or physical objects, and students know what constitutes source data and the various types of source data.  2. Teachers distribute medical records and case report forms. Students read the medical records and case report forms.  3. According to the medical records and case report forms prepared by the teacher, students should fill in:  (1) Case screening period (-7 ~ 0 days)  Inclusion criteria, exclusion criteria, inclusion determination, informed consent, demographic information, comorbidities at admission, onset and admission, signs and symptoms on admission, laboratory tests, other tests, medications taken before admission, and physical examination  (2) Treatment period (1-10 days) and subsequent stages (14-28 days)  Laboratory tests (D3, D7 and D10), other tests, concomitant drug record form, Adverse event (AE) record form and outcome |
| **Evaluate tab** |  |
| Simulation Evaluation | NA |
| **Debrief tab** |  |
| Guided reflection questions  (15 mins) | These guided reflection questions are organized by the gather-analyze-summarize (GAS) method. The questions are presented to suggest topics that may inspire the debriefing conversation. Gather Information………………………………………………………….3 mins  - How did you feel throughout the simulation experience? - What were the difficulties you encountered during this simulation? - Would one of you describe this simulation procedure from your perspective?  Analyze …………………………………………………………………...8 mins  - Describe the definition and related concepts of source data. - Describe the data collection and input process. - How did you overcome the difficulties encountered during the simulation?  Summarize …………………………………………………………………4 mins  - What did you learn from this experience? - What would you like to do differently next time in a similar situation? |

Class 10

Management and Report of Adverse Events

| **Overview tab** |  |
| --- | --- |
| Simulation Type | Short, didactic teaching and practical hands-on practice |
| Expected Theory Teaching Time | 30 minutes |
| Expected Simulation Run Time | 90 minutes |
| Guided Reflection Time | 15 minutes |
| Teacher’s Role | Instructors, facilitator |
| Student’s Simulation Role | Investigator, sponsor, CDE Health Commission, clinical trial institutions, IEC members |
| Simulated setting | Hospital |
| Learning Objectives | 1. Master: significance of safety evaluation; judgment, management and reporting of adverse events, adverse reactions, and serious adverse events.  2. Familiar with: ascertainment of adverse events, implementation of emergency unblinding.  3. Understand: coding of adverse events. |
| **Prepare tab** |  |
| Equipment List | Supplied by teachers   - PPT of theoretical knowledge - Adverse event evaluation standard CTCAE5.0 - Adverse event record form - Serious adverse event report form - Role cards   Supplied by students   - Emergency letters prepared in Class 7 - Laptop - Pen |
| Theory Teaching  (30 mins) | - Significance of safety evaluation - Definitions of common terms (adverse event, AE; adverse reaction; serious adverse event, SAE; serious adverse reaction; suspicious and unexpected serious adverse reactions, SUSA; development safety update report, DSUR) - MedDRA code - Evaluation of adverse events (severity, relevance to drugs) - Report of serious adverse events - Emergency unblinding |
| **Simulate tab** |  |
| Summary | Three simulated cases are given to determine whether adverse events have occurred, and if yes, the corresponding management and reporting of adverse events will be carried out. |
| Simulation case | Simulation case 1:  Random number: 002, name: Li Si, gender: male, date of birth: 19610212, ethnicity: Han. On the 7th day of follow-up after taking the medicine, total protein=41.5g/L, albumin=23.4g/L. |
|  | Simulation case 2:  Random number: 004, name: Zhang San, gender: male, date of birth: 19390629, ethnicity: Han. On the 10th day of follow-up after taking the medicine, multiple organ failure occurred, blood pressure was difficult to maintain and heart rate decreased. After a series of treatment measures, blood gas reexamination showed severe respiratory acidosis and metabolic acidosis. Finally, the rescue was ineffective and clinical death was declared. |
|  | Simulation case 3:  Random number: 005, name: Wang Wu, gender: female, date of birth: 19851205, nationality: Han. On the 20th day of follow-up after taking medicine, a car accident occurred on the road and she was diagnosed as having tibiofibular fracture. |
| Simulation operation (90 mins) | Judge the above three simulation cases:   - Is it an adverse event? - Is it a serious adverse event? - Is there any need for emergency unblinding? - If it is an adverse event, record it (SOC and PT from MedDRA software are preferred) - If it is a serious adverse event, groups are used to assign roles by themselves to simulate investigators, sponsors, CDE health committees, clinical trial institutions, and IEC members to report on serious adverse events. |
| **Evaluate tab** |  |
| Simulation Evaluation | Take the group as a unit to check the completion of the following contents, which are used for formative evaluation:   - Adverse event record form; - Serious adverse event report form (if required).   **The scoring is based on the below key points:**   - Right judgment of AE/SAE - Complete record and management process |
| **Debrief tab** |  |
| Guided reflection questions  (15 mins) | These guided reflection questions are organized by the gather-analyze-summarize (GAS) method. The questions are presented to suggest topics that may inspire the debriefing conversation. Gather Information………………………………………………………….3 mins  - How did you feel throughout the simulation experience? - What were the difficulties you encountered during this simulation? - Would one of you give a brief summary of this simulation?  Analyze …………………………………………………………………...8 mins  - Describe the definition of AE and SAE. How do you judge whether the simulated case is an AE/SAE? - What details should be recorded for AE and how did you get them? - What is the reporting process for SAE? - How did you cooperate within the group?  Summarize …………………………………………………………………4 mins  - What did you learn from this experience? - What would you like to do differently next time in a similar situation? |

Class 11

Unblinding and Statistical Analysis

| **Overview tab** |  |
| --- | --- |
| Simulation Type | Short, didactic teaching and practical hands-on practice |
| Expected Theory Teaching Time | 30 minutes |
| Expected Simulation Run Time | 90 minutes |
| Guided Reflection Time | 15 minutes |
| Teacher’s Role | Instructors, facilitator |
| Student’s Simulation Role | Statistician |
| Simulated setting | Program office |
| Learning Objectives | 1. Know the significance of unblinding and interpret the statistical results correctly.  2. Become familiar with: the specific steps of unblinding and the selection points for statistical methods.  3. Understand: procedures, statistical analysis plan and statistical analysis report. |
| **Prepare tab** |  |
| Equipment List | Supplied by teachers   - PPT of theoretical knowledge - Simulation database (electronic version)   Supplied by students   - Laptop with SPSS |
| Theory Teaching  (30 mins) | - Concept of unblinding - Significance of unblinding - Concept of common statistical methods of RCT, including statistical description and statistical inference - Operational process of statistical analysis |
| **Simulate tab** |  |
| Summary | Students play the role of statisticians for unblinding, use SPSS software to analyze the simulation database of RCT and interpret the results. |
| Simulation operation (90 mins) | 1.        First unblinding.  Determine the grouping of participates.  2.        Data cleaning.  Organize the database and define the types of variables according to the format requirements of SPSS software.  3.      Statistical description of baseline data.  Use Chi square test to test whether the difference between the two groups was statistically significant for categorical variables; Use T test or non-parametric test to test whether the difference between the two groups was statistically significant for continuous variables.  4.       Effectiveness and safety analysis.  Use Chi square test to analyze the primary outcome indicators between the two groups; Use Chi square test to analyze the safe outcome indicators between the two groups  5. Second unblinding.  Determine the drug taken by the participants. |
| **Evaluate tab** |  |
| Simulation Evaluation | The statistical analysis report files that record the results of statistical testing submitted in class by each student are used for formative evaluation.  **The scoring is based on the below key points:**   - Correct choice of statistical method - Correct statistical results - Correct interpretation of results |
| **Debrief tab** |  |
| Guided reflection questions  (15 mins) | These guided reflection questions are organized by the gather-analyze-summarize (GAS) method. The questions are presented to suggest topics that may inspire the debriefing conversation. Gather Information………………………………………………………….3 mins  - How did you feel throughout the simulation experience? - What were the difficulties you encountered during this simulation? - Would one of you describe this simulation procedure from your perspective?  Analyze …………………………………………………………………...8 mins  - Describe the definition of unblinding. How is unblinding achieved during the simulation? - Describe the contents of statistical analysis reports. Why do we select the corresponding statistical methods? How will you interpret the clinical significance for the statistical results? Will you be able to reproduce the results from the last time by doing it again? - How did you overcome the difficulties encountered during the simulation?  Summarize …………………………………………………………………4 mins  - What did you learn from this experience? - What would you like to do differently next time in a similar situation? |
