# Supplementary material for: Clinical research capability enhanced for medical undergraduates: an innovative simulation-based clinical research curriculum development
Source: BMC Med Educ. 2022 Jul 14;22:543. doi: 10.1186/s12909-022-03574-6 (PMC9281572; doi:10.1186/s12909-022-03574-6)
Supplement: Supplementary file 5 — Additional file 5. Simulation Class Design Scenario Script of Clinical Research 2 (ACross-sectional Study Simulation) [file 12909_2022_3574_MOESM5_ESM.docx]

Supplementary file 5

Simulation Design Scenario Script

A Cross-sectional Study Simulation

| **Field** | **Text** |
| --- | --- |
| Simulation Type | Short, didactic teaching and practical hands-on practice |
| Discipline | Clinical Medicine |
| Student Grade | Undergraduate |
| Simulation Research design | Cross-sectional study |
| Simulation Disease | Hypertension |
| Simulation Site | Communities in Wuhan, China |
| Simulation Mechanism/role | Study designer, investigator (mainly), statistician, monitor, subject |
| Simulation Case | Adults aged ≥ 18 years old resided in Wuhan |
| Location | Discussion Classroom |
| Learning strategies | Group Learning |

Contents

| **Class Number** | **Class Content** | Duration  (min) |
| --- | --- | --- |
| 1 | Writing a Cross-Sectional Study Protocol I | 3×45 |
| 2 | Writing a Cross-Sectional Study Protocol II | 3×45 |
| 3 | Development of Implementation Manual | 3×45 |
| 4 | Project Kick-Off Meeting | 3×45 |
| 5 | Field Investigation Workflow (Pilot Investigation, Field Investigation) | 3×45 |
| 6 | Data Management | 3×45 |
| 7 | Statistical Analysis of Cross-Sectional Study I | 3×45 |
| 8 | Statistical Analysis of Cross-Sectional Study II | 3×45 |
| 9 | Curriculum Extension: From Cross-Sectional to Longitudinal Study | 3×45 |
| 10 | Writing of Clinical Research Report | 3×45 |
| 11 | Oral Defense | 3×45 |

Class 1

Writing A Cross-Sectional Study Protocol I

| **Overview tab** |  |
| --- | --- |
| Simulation Type | Short, didactic teaching and practical hands-on practice |
| Expected Theory Teaching Time | 20 minutes |
| Expected Simulation Run Time | 100 minutes |
| Guided Reflection Time | 15 minutes |
| Teacher’s Role | Instructors, facilitator |
| Student’s Simulation Role | Study designer |
| Simulated setting | Program Office |
| Learning Objectives | 1. Become familiar with: the general principles for writing cross-sectional study protocols, preparation prior to drafting study protocol.  2. Understand: the key points for writing cross-sectional study protocol. |
| **Prepare tab** |  |
| Equipment List | Supplied by teachers   - PPT of theoretical knowledge - Cross-sectional study protocol template for hypertension investigation - STROBE (strengthening the reporting of observational studies in epidemiology) statement - Two published papers of cross-sectional studies   Supplied by students   - Laptop - Pen - Blank paper |
| Theory Teaching  (20 mins) | - Concept and purpose of cross-sectional studies - Main components of cross-sectional study protocols - Key points for writing study protocols - STROBE statement |
| **Simulate tab** |  |
| Summary | Students read the two published papers on cross-sectional studies, and write their own cross-sectional study protocol on the hypertension investigation in Wuhan, China (Chinese version). |
| Simulation operation (100 mins) | 1. Read the two articles “Protocol for the China PEACE (Patients-centered Evaluative Assessment of Cardiac Events) Million Persons Project pilot” (English version) and “Prevalence of hypertension and diabetes and the awareness rate of chronic disease risk factors in residents of Wuhan” (Chinese version).  2. Write the cross-sectional study protocol on the hypertension investigation in Wuhan, China, including:   - The background of the cross-sectional study on hypertension investigation. - The study objectives of of the cross-sectional study on hypertension investigation. - Inclusion and exclusion criteria according to the purpose of the research, and determine the target population. - The time period for conducting the investigation. - Research design type. - Sample size estimation (! Note: The content will be filled in next class). - Random sampling procedure. - The contents and methods of investigation. (! Note: Questionnaire design in the content will be filled in next class). - Quality control in the cross-sectional study. |
| **Evaluate tab** |  |
| Simulation Evaluation | The submitted protocol is used for formative evaluation for each student.  **The scoring is based on the key points below:**   - Structure, completeness and logicality - Protocol summary |
| **Debrief tab** |  |
| Guided reflection questions  (15 mins) | These guided reflection questions are organized by the gather-analyze-summarize (GAS) method. The questions are presented to suggest topics that may inspire the debriefing conversation. Gather Information………………………………………………………….4 mins  - How did you feel throughout the simulation experience? - What were the difficulties you encountered during this simulation? - Would one of you describe this simulation procedure from your perspective?  Analyze …………………………………………………………………...8 mins  - Describe the importance and necessity of a study protocol. - Describe the components of a cross-sectional study protocol. - How did you overcome the difficulties encountered during the simulation?  Summarize …………………………………………………………………3 mins  - What did you learn from this experience? - What would you like to do differently next time in a similar situation? |

Class 2

Writing A Cross-Sectional Study Protocol II

| **Overview tab** |  |
| --- | --- |
| Simulation Type | Short, didactic teaching and practical hands-on practice |
| Expected Theory Teaching Time | 35 minutes |
| Expected Simulation Run Time | 85 minutes |
| Guided Reflection Time | 15 minutes |
| Teacher’s Role | Instructors, facilitator |
| Student’s Simulation Role | Study designer |
| Simulated setting | Program office |
| Learning Objectives | 1. Grasp the evaluation of the reliability and validity of the questionnaire.  2. Become familiar with: the information needing to be collected during the investigation.  3. Understand: the estimation method for sample size, the influential factors for sample size, and questionnaire design. |
| **Prepare tab** |  |
| Equipment List | Supplied by teachers   - PPT of theoretical knowledge - Questionnaire template for the cross-sectional study on hypertension investigation   Supplied by students   - Laptop - Pen - Blank paper |
| Theory Teaching  (35 mins) | - Introduce the factors to be considered during the sample size estimation in the cross-sectional study - Introduce the sample size estimation methods in the cross-sectional study - Present the the sample size estimations for binary outcomes and continuous outcomes in cross-sectional studies. - Introduce the information needing to be collected during the investigation, the types and basic structure of questionnaires, and the method for questionnaire design - Introduce the evaluation of the reliability and validity of questionnaires |
| **Simulate tab** |  |
| **Scenario 1** | **Sample size estimation** |
| Teacher’s Role | Facilitator |
| Student’s Simulation Role | Study designer |
| Simulation operation (15 mins) | 1. According to the study objective of the cross-sectional study on hypertension investigation, students select the proper formula for sample size estimation.  2. Based on the given parameters from the published paper “Prevalence of hypertension and diabetes and the awareness rate of chronic disease risk factors in residents of Wuhan”, students use the formula to calculate the optimal sample size required for the cross-sectional study on hypertension investigation. |
| **Scenario 2** | **Questionnaire design** |
| Teacher’s Role | Facilitator |
| Student’s Simulation Role | Study designer |
| Simulation operation (70 mins) | Design the questionnaire of cross-sectional study on hypertension investigation, including:   - Cover letter including an introduction to the program, a brief summary of what will be with the collected data - General instructions for filling the questionnaire - Basic information of the respondents - Information on the characteristics associated with hypertension |
| **Evaluate tab** |  |
| Simulation Evaluation | The estimated sample size with specified parameters and questionnaire for the cross-sectional study on hypertension investigation are used for formative evaluation for each student.  **The scoring is based on the key points below:**   - Proper results for sample size estimation - Appropriate questionnaire for the cross-sectional study on hypertension investigation |
| **Debrief tab** |  |
| Guided reflection questions  (15 mins) | These guided reflection questions are organized by the gather-analyze-summarize (GAS) method. The questions are presented to suggest topics that may inspire the debriefing conversation. Gather Information………………………………………………………….3 mins  - How did you feel throughout the simulation experience? - What were the difficulties you encountered during this simulation? - Would one of you give a brief summary of this simulation?  Analyze …………………………………………………………………...8 mins  - Describe the importance and necessity of sample size estimation. - Describe the required parameters in sample size estimation. - Describe the structure of the questionnaire.  Summarize …………………………………………………………………4 mins  - What did you learn from this experience? - What would you like to do differently next time in a similar situation? |

Class 3

Development of Implementation Manual

| **Overview tab** |  |
| --- | --- |
| Simulation Type | Short, didactic teaching and practical hands-on practice |
| Expected Theory Teaching Time | 30 minutes |
| Expected Simulation Run Time | 90 minutes |
| Guided Reflection Time | 15 minutes |
| Teacher’s Role | Instructors, facilitator |
| Student’s Simulation Role | Study designer |
| Simulated setting | Program office |
| Learning Objectives | 1.Master: the methods for the development of an implementation manual for “Questionnaire on hypertension in Wuhan, Hubei Province, China”, the content of implementation manual such as investigation members, organization structure of investigation, common problems that may occur on site and relevant emergency treatments, etc.  2.Become familiar with: the purpose and importance of the implementation manual. |
| **Prepare tab** |  |
| Equipment List | Supplied by teachers   - PPT of theoretical knowledge - Reference template of implementation manual - Manual of procedures of Framingham Heart Study - Manual of procedures of China Health and Retirement Longitudinal Study - Questionnaire on hypertension in Wuhan, Hubei Province, China - Implementation manual for “Questionnaire on hypertension in Wuhan, Hubei Province, China”   Supplied by students   - Laptop - Pen - Blank paper |
| Theory Teaching  (30 mins) | - Introduce the purpose and importance of the implementation manual - Teach how to formulate the implementation manual, including manufacturing procedures and basic elements - Introduce basic contents and precautions of the questionnaire on hypertension in Wuhan, Hubei Province, China |
| **Simulate tab** |  |
| Simulation operation (90 mins) | 1. Teachers will distribute “reference template of implementation manual”, “Manual of procedures of Framingham Heart Study”, “Manual of procedures of China Health and Retirement Longitudinal Study”, and “Questionnaire on Hypertension in Wuhan, Hubei Province, China”.  2. Under the guidance of teachers, students should carefully read and understand the characteristics of reference template of the implementation manual, manual of procedures of Framingham Heart Study or China Health and Retirement Longitudinal Study and the basic contents of the questionnaire on hypertension in Wuhan, Hubei Province, China.  3. According to the grouping arrangement, the students in each group will select 2 ~ 3 questions for each topic and set up 2 ~ 3 on-site common problems and emergency treatments based on the basic information, lifestyle, disease history and family history, anthropometry and laboratory tests involved in the questionnaire on hypertension in Wuhan, Hubei Province, China. The students will try to develop the implementation manual of the questionnaire. One lecturer and four assistants provide guidance in class.  4. Teachers will distribute implementation manual for “Questionnaire on hypertension in Wuhan, Hubei Province, China”.  5. Each group leader will report the contents of the implementation manual formulated by the group and the problems encountered. Each group will take 10 ~ 15 minutes. One lecturer and four assistants will randomly ask questions and answer relevant questions.  6. Teachers will summarize the content of courses and explain the precautions. Students can ask questions and participate in the interaction. |
| **Evaluate tab** |  |
| Simulation Evaluation | The implementation manuals formulated by the group are used for formative evaluation.  **The scoring is based on the below key points:**   - Structure - Contents |
| **Debrief tab** |  |
| Guided reflection questions  (15 mins) | These guided reflection questions are organized by the gather-analyze-summarize (GAS) method. The questions are presented to suggest topics that may inspire the debriefing conversation. Gather Information………………………………………………………….3 mins  - How did you feel throughout the simulation experience? - What were the difficulties you encountered during this simulation? - Would one of you give a brief summary of this simulation?  Analyze …………………………………………………………………...8 mins  - Describe the purpose and importance of the implementation manual. - Describe the methods and characteristics for the development of implementation manuals.  Summarize …………………………………………………………………4 mins  - What did you learn from this experience? - What would you like to do differently next time in a similar situation? |

Class 4

Project Kick-Off Meeting (Project Training)

| **Overview tab** |  |
| --- | --- |
| Simulation Type | Short, didactic teaching and practical hands-on practice |
| Expected Theory Teaching Time | 30 minutes |
| Expected Simulation Run Time | 90 minutes |
| Guided Reflection Time | 15 minutes |
| Teacher’s Role | Instructors, facilitator |
| Student’s Simulation Role | Investigator |
| Simulated setting | Meeting room |
| Learning Objectives | 1. Learn the overall process of the investigation of hypertension in Wuchang District, Wuhan City, Hubei Province. 2. Become familiar with: preliminary preparations and precautions for field investigation. 3. Understand: training process and precautions for various participants. |
| **Prepare tab** |  |
| Equipment List | Supplied by teachers   - PPT of theoretical knowledge - Registration form for family members - Appointment letter - Field investigation registration form - Informed Consent Form - Hypertension in Wuchang District, Wuhan City, Hubei Province survey questionnaire - Field investigation transfer form - Survey result feedback sheet - Height and weight measuring instrument - Electronic sphygmomanometer - Nylon tape measure   Supplied by students   - Paper and pen - Implementation Manual made in last class |
| Theory Teaching  (30 mins) | - Review the overall process of the investigation of the current situation of hypertension in Wuchang District, Wuhan City, Hubei Province - Introduce the preliminary preparations and points of attention for field investigation - Introduce the purpose, significance, and content of the project kick-off meeting and project training |
| **Simulate tab** |  |
| Summary | The teacher will give a demonstration first, and then the students will play roles in the group to simulate the training process of informed consent investigators, questionnaire investigators, and physical examination investigators. |
| Simulation operation (90 mins) | 1. The teacher will randomly select one student from each group who will then make a presentation:   - For training leaders, give them a detailed understanding of the overall process of the project; - For others, train investigators in a specific area of the work, including informed consent investigators, questionnaire investigators, and physical examination investigators.   2. Students in each group simulate the training process of the project:   1. The students will be divided into 6 groups with 7 students in each group: 1 leader, 2 informed consent investigators, 2 questionnaire investigators, and 2 physical examination investigators. 2. Training of informed consent investigators:  - The content of informed consent. - In the process of informed consent, the mother tongue of the respondents should be used, and professional terms should be avoided as far as possible. - To ensure the respondents have understood all aspects of e informed consent. - Respondents must be given sufficient time to consider whether they want to participate in this research. - For those who are unable to give consent, the explanation of informed consent shall be provided to their legal representatives. - The respondents should not be coerced or improperly influenced to make a decision on whether to participate in the research project. - The informed consent should be signed and dated by the investigation subject or his legal representative.  1. Training of questionnaire investigators:  - Emphasized the uniform standards. - The attitude should be sincere, and amiable while administering the questionnaire. - Pay attention to the respondents psychological feelings during the questionnaire administeration. - Method of filling in the questionnaire: it is best for the investigator to fill in the questionnaire as respondents answer the questions. - The investigator should be familiar with the questionnaire and understand the purpose of each question. - Use words that are easy to understand.  1. Training of physical examination investigators:  - Measurement of height - Measurement of weight - Measurement of waist circumference - Measurement of blood pressure  1. Record the problems in the training process, and unified standards within the group. |
| **Evaluate tab** |  |
| Simulation Evaluation | NA |
| **Debrief tab** |  |
| Guided reflection questions  (15 mins) | These guided reflection questions are organized by the gather-analyze-summarize (GAS) method. The questions are presented to suggest topics that may inspire the debriefing conversation. Gather Information………………………………………………………….4 mins  - How did you feel throughout the simulation experience? - What were the difficulties you encountered during this simulation? - Would one of you describe this simulation procedure from your perspective?  Analyze …………………………………………………………………...8 mins  - Describe the components in a project kick-off meeting (or project training). - How did you overcome the difficulties encountered during the simulation?  Summarize …………………………………………………………………3 mins  - What did you learn from this experience? - What would you like to do differently next time in a similar situation? |

Class 5

Field Investigation Workflow (Pilot Investigation, Field Investigation)

| **Overview tab** |  |
| --- | --- |
| Simulation Type | Short, didactic teaching and practical hands-on practice |
| Expected Theory Teaching Time | 30 minutes |
| Expected Simulation Run Time | 90 minutes |
| Guided Reflection Time | 15 minutes |
| Teacher’s Role | Instructors, facilitator |
| Student’s Simulation Role | Investigator, community resident |
| Simulated setting | Household of community resident, Community health center |
| Learning Objectives | 1. Describe the content and workflow of field investigation.  2. Be familiar with the quality control and principles of the field investigation.  3. Understand the purpose, significance and method of the pilot investigation. |
| **Prepare tab** |  |
| Equipment List | Supplied by teachers   - PPT of theoretical knowledge - Family member registration sheet - Appointment letter - Field investigation registration sheet - Informed consent sheet - Questionnaire - Investigation transfer sheet - Investigation result feedback sheet - Height and weight measuring instrument - Electronic sphygmomanometer - Blood glucose meter - Blood sample test tube   Supplied by students   - Paper and Pencil |
| Theory Teaching  (30 mins) | - Introduce the purpose, significance and method of the pilot survey - Introduce the content and procedure of field investigation - Introduce the quality control and principles of the field investigation |
| **Simulate tab** |  |
| **Scenario 1** | **Appointment of study subjects** |
| Teacher’s Role | Facilitator |
| Student’s Simulation Role | Investigator, community resident |
| Simulation operation (45mins) | Taking the group as the unit, the investigator and community resident will be simulated respectively. According to the content of subject appointment, students simulate the appointment process in the home of community resident.  The following two situations should be included in the simulation：   - One family agreed to participate with one family member who did not meet the inclusion and exclusion criteria - One family refused to participate |
| **Scenario 2** | **Field investigation** |
| Teacher’s Role | Facilitator |
| Student’s Simulation Role | Investigator, community resident |
| Simulation operation (45mins) | Taking the group as the unit, the investigator and community resident will be simulated respectively. According to the content and workflow of field investigation, students simulate the field investigation at the community health center:   - **Identity verification:** check whether the appointment letter, ID card of the resident are consistent with the information of the resident in the field investigation registration sheet. - **Informed consent:** after the identity verification, the informed consent sheet is assigned to each resident, and the investigation transfer sheet is given to the resident. - **Questionnaire survey:** complete the questionnaire through inquiry. - **Body measurement:** measure the height, weight, waist circumference and blood pressure of the resident. - **Blood sample collection:** collect the resident’s blood sample and the investigation transfer sheet. |
| **Evaluate tab** |  |
| Simulation Evaluation | NA |
| **Debrief tab** |  |
| Guided reflection questions  (15 mins) | These guided reflection questions are organized by the gather-analyze-summarize (GAS) method. The questions are presented to suggest topics that may inspire the debriefing conversation. Gather Information………………………………………………………….4 mins  - How did you feel throughout the simulation experience? - What were the difficulties you encountered during this simulation?  What could you do improve the willingness of the resident to participate?Analyze …………………………………………………………………...8 mins  - Describe the factors that would influence the willingness of a resident to participate. - Describe the workflow of a field investigation. - How did you overcome the difficulties encountered during the simulation?  Summarize …………………………………………………………………3 mins  - What did you learn from this experience? - What would you like to do differently next time in a similar situation? |

Class 6

Data Management

| **Overview tab** |  |
| --- | --- |
| Simulation Type | Short, didactic teaching and practical hands-on practice |
| Expected Theory Teaching Time | 30 minutes |
| Expected Simulation Run Time | 90 minutes |
| Guided Reflection Time | 15 minutes |
| Teacher’s Role | Instructors, facilitator |
| Student’s Simulation Role | Statistician |
| Simulated setting | Program office |
| Learning Objectives | 1. Become familiar with: database establishment and data entry by Epidata software.  2. Describe the content of data verification and know how to undertake data verification, write data questioning document and deal with problem data.  3. Understand the significance and importance of data management. |
| **Prepare tab** |  |
| Equipment List | Supplied by teachers   - PPT of theoretical knowledge - Epidata software, software operation tutorial and demonstration files - Questionnaire on hypertension investigation - Simulation database - Sample data questioning file - Feedback of problem data   Supplied by students   - Laptop |
| Theory Teaching  (30 mins) | - Definition and meaning of data management - Establish of database - Data collection methods (including paper version data collection + computer transcription, electronic data collection), Electronic Data Capture (EDC) System - Content and method of data verification - Data confidentiality and security |
| **Simulate tab** |  |
| Summary | According to the hypertension questionnaire made in Class 2, establish the database and input the simulated data. Check the simulated data, write the data questioning document and deal with problem data according to the feedback. |
| Simulation operation (90 mins) | 1. Database establishment and data entry by Epidata software  Establish database, including naming of variables and coding of answers.   - The teacher demonstrates the operation of Epidata software, displays the QES file to establish database, generates the REC file according to the QES file, and establishes the CHK verification file. - Students simulate building an EpiData database based on the first two pages of the hypertension questionnaire, and save QES, blank REC files or necessary CHK file, based on the teacher's demonstration and software operation tutorial. - Students simulate entering a record (i.e. the information of the first subject), according to the simulation data provided by the teacher, and export the data   2. Data verification based on the simulation data   - Write a data verification plan (DVP) and describe the main contents of data verification - Conduct data verification and write a data questioning file - Deal with problem data according to teachers’ feedback |
| **Evaluate tab** |  |
| Simulation Evaluation | - The following files are used for formative evaluation for each student. - QES, REC files or necessary CHK file related to database establishment and data entry - Data verification plan - Data questioning file - Data after verification   **The scoring is based on the key points below:**   - Correct - Complete - Standardized |
| **Debrief tab** |  |
| Guided reflection questions  (15 mins) | These guided reflection questions are organized by the gather-analyze-summarize (GAS) method. The questions are presented to suggest topics that may inspire the debriefing conversation. Gather Information………………………………………………………….3 mins  - How did you feel throughout the simulation experience? - What were the difficulties you encountered during this simulation? - Would one of you give a brief summary of this simulation?  Analyze …………………………………………………………………...8 mins  - What do you think are the key points when establishing a database? - Describe the common data problems during data verification.  Summarize …………………………………………………………………4 mins  - What did you learn from this experience? - What would you like to do differently next time in a similar situation? |

Class 7

Statistical Analysis of Cross-Sectional Study I

| **Overview tab** |  |
| --- | --- |
| Simulation Type | Short, didactic teaching and practical hands-on practice |
| Expected Theory Teaching Time | 30 minutes |
| Expected Simulation Run Time | 90 minutes |
| Guided Reflection Time | 15 minutes |
| Teacher’s Role | Instructors, facilitator |
| Student’s Simulation Role | Statistician |
| Simulated setting | Program office |
| Learning Objectives | 1. Master: The application conditions and interpretation of analysis results of common statistical methods in cross-sectional studies.  2.Be familiarize with: The specific steps and the selection points for statistical methods in cross-sectional studies.  3. Understand: The principles of statistical analysis methods used in cross-section studies. |
| **Prepare tab** |  |
| Equipment List | Supplied by teachers   - PPT of theoretical knowledge - Simulation database (electronic version)   Supplied by students   - Laptop with installed SPSS software |
| Theory Teaching  (30 mins) | - Concept of common statistical methods for cross-section studies, including statistical description and t test, chi-square test, nonparametric test, logistic regression analysis - Operational process of these statistical analysis methods |
| **Simulate tab** |  |
| Summary | Students play the role of statisticians while using SPSS software to analyze the database of cross-section studies and interpret the results. |
| Simulation operation (90 mins) | 1.       The establishment of SPSS database and data cleaning.  Import Excel file and change the labels in SPSS variable view; Organize the database and define the types of variables according to the format requirements of SPSS software.  2.        Statistical description.  Use Mean±SD to describe the characteristics of continuous variables; Use case and frequency to display the characteristics of categorical variables.  3.     t test, chi-square test, nonparametric test, logistic regression analysis.  Use different statistical methods to analyze the SPSS database.  4. Interpretation of the analysis results.  Explain the clinical significance of the different statistical results. |
| **Evaluate tab** |  |
| Simulation Evaluation | The statistical analysis report files that record the results of statistical tests submitted in class by each student are used for formative evaluation.  **The scoring is based on the below key points:**   - Correct choice of statistical method - Correct statistical results - Correct interpretation of results |
| **Debrief tab** |  |
| Guided reflection questions  (15 mins) | These guided reflection questions are organized by the gather-analyze-summarize (GAS) method. The questions are presented to suggest topics that may inspire the debriefing conversation. Gather Information………………………………………………………….3 mins  - How did you feel throughout the simulation experience? - What were the difficulties you encountered during this simulation? - Would one of you describe this simulation procedure from your perspective?  Analyze …………………………………………………………………...8 mins  - Describe the contents of statistical analysis reports. - Why do we select the corresponding statistical methods? - How will you interpret the clinical significance for the statistical results? - Will you be able to reproduce the results from the last time by doing it again? - How did you overcome the difficulties encountered during the simulation?  Summarize …………………………………………………………………4 mins  - What did you learn from this experience? - What would you like to do differently next time in a similar situation? |

Class 8

Statistical Analysis of Cross-Sectional Study II

| **Overview tab** |  |
| --- | --- |
| Simulation Type | Short, didactic teaching and practical hands-on practice |
| Expected Theory Teaching Time | 30 minutes |
| Expected Simulation Run Time | 90 minutes |
| Guided Reflection Time | 15 minutes |
| Teacher’s Role | Instructors, facilitator |
| Student’s Simulation Role | Statistician |
| Simulated setting | Statistical programming department |
| Learning Objectives | 1. Master: descriptive analysis and hypothesis testing (prevalence, univariable analyses and multivariable regression)  2. Be familiar with: distribution of disease (who, where, when), properties and limitations of effect measures and conclusions in cross-sectional studies  3. Understand: objectives, meanings and methods of statistical analysis for cross-sectional studies. |
| **Prepare tab** |  |
| Equipment List | Supplied by teachers   - PPT of theoretical knowledge - Simulation data for analysis practice - Table and figure template for summarizing results - STROBE guideline and checklist - Reporting guideline for cross-sectional study - The SAMPL guidelines - Two examples using cross-sectional design   Supplied by students   - Laptop with installed SPSS software |
| Theory Teaching  (30 mins) | - Introduce purposes, components and evaluation of analysis practice - Introduce STROBE, SAMPL and Reporting guideline for cross-sectional studies - Introduce structure of simulation data and reporting template |
| **Simulate tab** |  |
| Summary | Students summarize required analytical methods in cross-sectional studies from guidelines and examples, and then analyze the simulation data using SPSS software and complete the reporting template. |
| Simulation operation (90 mins) | 1.Students learn methods presented in the SAMPL guidelines, reporting guideline for cross-sectional studies.  2.Students read the two examples and summarize required analytical methods.  3.Students analyze the simulation data using SPSS software and complete the reporting template, including:   - flowchart (inclusion and exclusion) - descriptive analysis - prevalence of hypertension and 95% confidence interval in total sample and between different characteristics. - univariable and multivariable logistic regression to explore associated risk factors. |
| **Evaluate tab** |  |
| Simulation Evaluation | The statistical report is used for evaluation for formative evaluation.  **The scoring is based on the below key points:**   - Clear inclusion and exclusion in the flowchart - Appropriate results in tables |
| **Debrief tab** |  |
| Guided reflection questions  (15 mins) | These guided reflection questions are organized by the gather-analyze-summarize (GAS) method. The questions are presented to suggest topics that may inspire the debriefing conversation. Gather Information………………………………………………………….4 mins  - How do you feel throughout the simulation experience? - What are the difficulties you encountered during this simulation? - Would you describe this simulation procedure from your perspective?  Analyze …………………………………………………………………...8 mins  - Describe the properties of cross-sectional studies. - Describe the components of statistical templates. - How do you overcome the difficulties encountered during the simulation?  Summarize …………………………………………………………………3 mins  - What do you learn from this experience? - What would you like to do differently next time in a similar situation? |

Class 9

Curriculum Expansion: From Cross-Sectional to Longitudinal Study

| **Overview tab** |  |
| --- | --- |
| Simulation Type | Short, didactic teaching and practical hands-on practice |
| Expected Theory Teaching Time | 45 minutes |
| Expected Simulation Run Time | 75 minutes |
| Guided Reflection Time | 15 minutes |
| Teacher’s Role | Instructors, facilitator |
| Student’s Simulation Role | Statisticians |
| Simulated setting | Statistical programming department |
| Learning Objectives | 1. Master: difference in objectives, advantage and disadvantage between cross-sectional and longitudinal studies; follow-up, effect measures and common statistical methods in longitudinal studies.  2. Familiar with: design type and outcomes in longitudinal studies  3. Understand: causal inference in longitudinal studies |
| **Prepare tab** |  |
| Equipment List | Supplied by teachers   - PPT of theoretical knowledge - Simulation data for analysis practice - Table and figure template for summarizing results - STROBE guideline and checklist (cohort study section) - Statistical analysis framework and common statistical analysis methods in medical research - Two examples using cohort design   Supplied by students   - Laptop with installed SPSS software |
| Theory Teaching  (45 mins) | - Causal inference and common rules - Limitation of cross-sectional study in casual relationship in disease - Property and common designs in longitudinal study - Common conception and statistical analysis (follow-up, incidence, survival analysis) - Cohort study and structure of simulation data |
| **Simulate tab** |  |
| Simulation operation (75 mins) | 1.Students learn methods presented in the SROBE guidelines, Statistical analysis framework and common statistical analysis methods.  2.Students read the two examples and summarize required analytical methods  3.Students analyze the simulation data using SPSS software and complete the reporting template, including:   - flowchart (inclusion and exclusion) - descriptive analysis and group comparison - Draw Kaplan-Meier survival curve and conduct log-rank test - Univariable and multivariable COX regression - Summarize results and write statistical reports |
| **Evaluate tab** |  |
| Evaluation basis | The statistical report is used for formative evaluation.  **The scoring is based on the key points below:**   - Clear inclusion and exclusion in the flowchart - Appropriate KM plot and results in tables |
| **Debrief tab** |  |
| Guided reflection questions  (15 mins) | These guided reflection questions are organized by the gather-analyze-summarize (GAS) method. The questions are presented to suggest topics that may inspire the debriefing conversation. Gather Information………………………………………………………….3 mins  - How did you feel throughout the simulation experience? - What were the difficulties you encountered during this simulation? - Would one of you give a brief summary of this simulation?  Analyze …………………………………………………………………...8 mins  - Describe the importance and necessity of longitudinal studies. - Describe the effect measures and common statistical methods in longitudinal methods.  Summarize …………………………………………………………………4 mins  - What did you learn from this experience? - What would you like to do differently next time in a similar situation? |

Class 10

Writing A Clinical Research Report

| **Overview tab** |  |
| --- | --- |
| Simulation Type | Short, didactic teaching and practice |
| Expected Theory Teaching Time | 30 minutes |
| Expected Simulation Run Time | 105 minutes |
| Teacher’s Role | Instructors, facilitator |
| Student’s Simulation Role | Investigator |
| Simulated setting | Office |
| Learning Objectives | Master the key points of the method of writing a clinical research report. |
| **Prepare tab** |  |
| Equipment List | Supplied by teachers   - Related published papers for reference - guideline and checklist for reporting clinical research, such as CONSORT statement, STROBE   Supplied by students   - Laptop |
| Theory Teaching  (30 mins) | Introduction of common guideline and checklist for reporting clinical research. |
| **Simulate tab** |  |
| Simulation operation (105 mins) | Summarize the entire simulation research process and write the report as a group according to the corresponding reporting guideline. |
| **Evaluate tab** |  |
| Evaluation basis | The writing of a research report is impossible to complete in class, so students should complete this in their spare time and prepare for the oral defense in next class as a group. |

Class 11

Oral Defense

| **Overview tab** |  |
| --- | --- |
| Simulation Type | Practice |
| Expected Simulation Run Time | 120 minutes |
| Guided Reflection Time | 15 minutes |
| Teacher’s Role | Defense teacher, facilitator |
| Student’s Simulation Role | Respondent |
| Simulated setting | Meeting room |
| Learning Objectives | Master the method of thesis report and defense. |
| **Prepare tab** |  |
| Equipment List | Supplied by teachers   - Thesis defense review forms - Draw lots   Supplied by students   - Printed paper - PPT for reporting - Laptop - Division of team members |
| **Simulate tab** |  |
| Simulation operation (120 mins) | 1. The group leaders draw lots to determine the reporting classroom and the reporting sequence of the group (5 minutes).  2. Copy the PPT for reporting to the classroom computer in advance and give the printed paper and division of team members to the defense teachers (15 minutes).  3. According to the order of drawing lots, each group will report in turn (20 minutes for each group).  4.Questions and comments from defense teachers and/or students from other groups, and responses of the reporting group (15 minutes for each group).  5.Teachers summarized the whole process. |
| **Evaluate tab** |  |
| Evaluation basis | The paper and presentation were used for summative evaluation.  **The scoring is based on the key points below:**   - The paper, 60%, including complete structure, correct method, reliable results, reasonable interpretation, etc. - The presentation, 40%, including the reporting time, reporting the main contents of the thesis fluently and clearly and answering questions accurately. |
| **Debrief tab** |  |
| Guided reflection questions  (15 mins) | These guided reflection questions are organized by the gather-analyze-summarize (GAS) method. The questions are presented to suggest topics that may inspire the debriefing conversation. Gather Information………………………………………………………….3 mins  - How did you feel throughout the simulation experience? - What were the difficulties you encountered during this simulation? - Would one of you give a brief summary of this simulation?  Analyze …………………………………………………………………...8 mins  - Summarize the key points of thesis writing. - Summarize the key points of oral defense.  Summarize …………………………………………………………………4 mins  - What did you learn from this experience? - What would you like to do differently next time in a similar situation? |
